# Engineering durable interphases for high-voltage Li-ion batteries under thermal stress

**DOI:** 10.1093/nsr/nwaf345

**Published:** 2025-08-21

**Authors:** Shiming Chen, Wenguang Zhao, Guorui Zheng, Chenyu Yang, Taowen Chen, Hengyu Ren, Yue Zuo, Xiangming Yao, Ke Li, Haoyu Xue, Jianjun Fang, Yuxiang Huang, Kai Yang, Zu-Wei Yin, Luyi Yang, Feng Pan

**Affiliations:** School of Advanced Materials, Peking University Shenzhen Graduate School, Shenzhen 518055, China; School of Advanced Materials, Peking University Shenzhen Graduate School, Shenzhen 518055, China; Institute of Materials Research (IMR), Tsinghua Shenzhen International Graduate School, Tsinghua University, Shenzhen 518055, China; National Synchrotron Radiation Laboratory, University of Science and Technology of China, Hefei 230029, China; School of Advanced Materials, Peking University Shenzhen Graduate School, Shenzhen 518055, China; School of Advanced Materials, Peking University Shenzhen Graduate School, Shenzhen 518055, China; School of Advanced Materials, Peking University Shenzhen Graduate School, Shenzhen 518055, China; School of Advanced Materials, Peking University Shenzhen Graduate School, Shenzhen 518055, China; School of Advanced Materials, Peking University Shenzhen Graduate School, Shenzhen 518055, China; School of Advanced Materials, Peking University Shenzhen Graduate School, Shenzhen 518055, China; School of Advanced Materials, Peking University Shenzhen Graduate School, Shenzhen 518055, China; Department of Chemistry, University of Hong Kong, Hong Kong 999077, China; Advanced Technology Institute, University of Surrey, Guildford GU2 7XH, UK; College of Energy, Xiamen University, Xiamen 361005, China; School of Advanced Materials, Peking University Shenzhen Graduate School, Shenzhen 518055, China; School of Advanced Materials, Peking University Shenzhen Graduate School, Shenzhen 518055, China

**Keywords:** LiCoO_2_ cathode, cathode electrolyte interphase, high-voltage and elevated temperature, electrolyte design strategy

## Abstract

Achieving stable cycling of high-voltage cathodes at elevated temperatures remains a critical challenge due to intensified interfacial side reactions and the accelerated dissolution of the cathode electrolyte interphase (CEI). In this work, based on theoretical calculations, Li_2_SO_3_ with desirable thermal stability and adhesiveness is proposed as a thermally stable binding agent between the selected CEI components (LiF and Li_3_PO_4_, where Li_3_PO_4_ facilitates fast Li^+^ transport and thermal stability, while LiF serves as a stable, electron-blocking framework) to prevent undesirable dissolution. This is achieved by leveraging electrolyte engineering to modulate the solvation environment and promote the *in-situ* formation of the aforementioned CEI architecture. Owing to its structural and compositional stability at elevated temperatures, the constructed CEI effectively mitigates interfacial side reactions even under elevated temperature conditions. As a result, the uncommon achievement of stable cycling for LiCoO_2_ at 4.6 V and 45°C was realized, demonstrating 81.9% capacity retention over 500 cycles. This work provides a transformative pathway for designing a durable CEI layer tailored to high-voltage and elevated temperature applications, paving the way for lithium-ion batteries to operate reliably in extreme environments.

## Teaser

This work presents a cathode electrolyte interphase design strategy with improved chemical and thermal stability through electrolyte engineering, achieving the stability of cathode materials under harsh conditions of high-voltage and elevated temperature.

## INTRODUCTION

Driven by the ever-increasing demand for high energy density in portable electronic devices and electric vehicles, there is a growing need for advanced cathode materials in lithium-ion batteries (LIBs) that can be cycled stably at high cutoff voltages while delivering large specific capacities [1,2]. However, extending the operating voltage beyond 4.5 V vs Li/Li⁺ poses significant challenges, including severe side reactions and irreversible phase transitions. Commercially used carbonate-based solvents are considered to form a non-uniform cathode electrolyte interphase (CEI), which is mainly composed of organic species with limited oxidative stability [3,4]. To stabilize the cathode interphase under high-voltage (HV) conditions, an effective strategy is to modify the electrolyte to construct a CEI layer with desirable physicochemical properties [5]. For instance, the introduction of lithium difluoro(oxalato)borate contributes to construction of a lithium fluoride (LiF)-rich interphase [6,7], which has been considered as an ideal inorganic component due to its chemical inertness, high Young's modulus and low electronic conductivity [8], thus suppressing degradation of the cathode. Besides, a suitable ion conductor such as lithium phosphate (Li_3_PO_4_, ∼10^−6^ S cm^−1^) is proved to reduce the redox activity for interfacial oxygen anions and suppress transition metal (TM) ion dissolution, effectively enhancing stability of the cathode [9,10].

Although the aforementioned efforts have successfully developed stable CEI layers on the cathode surface under HV conditions, few studies have simultaneously addressed cycling stability at elevated temperatures, which is a critical safety metric for commercial LIBs [11]. Under such harsh conditions, cathodes would suffer more severe interfacial issues, including excess electrolyte decomposition and CEI dissolution [12,13]. The organic species in the CEI layer tend to dissolve in the electrolyte at elevated temperature due to lack of thermal stability, resulting in the dense CEI structure to be destroyed at room temperature [14–16]. In this case, alternative CEI components need to be introduced, which not only exhibit a wide bandgap and high thermal stability, but also feature a robust adhesion interaction with coexisting inorganic components, thus maintaining the structural integrity of the CEI layer. In previous studies, the cycling stability of cathodes under elevated temperature conditions have not received much attention [16,17]. Considering the diverse application scenarios and safety requirements of LIBs, it is crucial to regulate the electrolyte composition and solvation structure and construct a stable CEI structure under such harsh conditions.

Herein, the essential physiochemical properties of inorganics in CEI required to perform effectively under extreme conditions were outlined, including band gap, ionic conductivity, thermal stability and adhesive force. Therefore, we propose a multiple inorganics strategy for the design for CEIs under harsh conditions, where Li_3_PO_4_ facilitates fast Li^+^ transport and high thermal stability, while lithium sulfite (Li_2_SO_3_) provides the necessary adhesiveness and LiF serves as a stable framework. Through introducing triethyl phosphate (TEP) and 1,3-propane sultone (PS) into the electrolyte, a trilayered CEI composed of Li_3_PO_4_, LiF and Li_2_SO_3_ was constructed on the lithium cobalt oxide (LiCoO_2_, LCO) cathode, which exhibits high chemical-electrochemical stability and excellent anchoring strength. The designed CEI layer could effectively suppress surface degradation of LCO and restrain detrimental side reactions under harsh testing conditions (4.6 V at 45°C). Benefitting from the construction of robust CEI on the cathode surface, high capacity and excellent cycling stability have been achieved, significantly outperforming traditional carbonate-based electrolytes.

## RESULTS AND DISCUSSION

### Design principles and physicochemical properties of electrolytes

**Figure 1.**
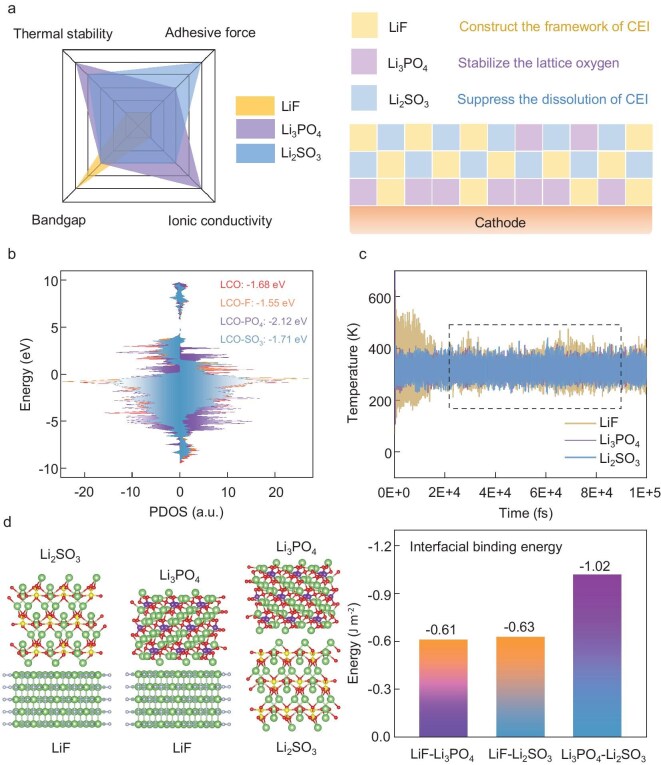
(a) Radar charts of three inorganic components and schematic illustration of CEI design. The bandgap and ionic conductivity data are collected from the previous research and material database [38–42]; (b) calculated density of states of O 2p for the outermost single layer of cobalt-oxygen in LCO with various functional groups; (c) temperature curves of LiF, Li_3_PO_4_ and Li_2_SO_3_ in AIMD calculation at 45°C (the more the curve fluctuation the more unstable the component); (d) interfacial binding energies between the LiF (100), Li_3_PO_4_ (101) and Li_2_SO_3_ (010).

As shown in Fig. 1a, although LiF, as a major component of the CEI, has a relatively wide electrochemical window, its properties do not meet the aforementioned requirements in other aspects. During the deep de-lithiation process, the LCO cathode tends to suffer from the escape of active oxygen species and the release of low-valence TM ions in order to maintain charge balance on the surface [18]. This process disrupts the CEI layers and exacerbates interface reactions. It is efficient to lower the O *2p* band center of the LCO cathode, thereby improving structural and interfacial stability [19]. Combined with the results of partial density of states (PDOS) for de-lithiated LCO cathodes, the role of various anions (F^−^, PO_4_^3−^ and SO_3_^2−^) in enhancing interfacial oxygen stability at the atomic level are revealed (Fig. 1b and Fig. S1). Compared with a pristine LCO cathode, both PO_4_^3−^ and SO_3_^2−^ reduce the O 2p band center position (−2.12 eV for LCO-PO_4_ and −1.71 eV for LCO-SO_3_), indicating a significant suppression of redox activity for oxygen anions.

Furthermore, to preserve the CEI integrity under elevated temperature conditions, the newly incorporated species should feature excellent thermal resilience and robust adhesion with coexisting interphase components. The thermal stability of various CEI components was evaluated by density functional theory (DFT) calculation and *ab initio* molecular dynamics (AIMD) simulation. Li_3_PO_4_ shows the largest cohesive energy (indicating the degree of interaction and bond strength in the interior of the material) due to the robust P-O tetrahedron groups (Fig. S2). The temperature curves of LiF, Li_3_PO_4_ and Li_2_SO_3_ obtained from AIMD simulation show that Li_2_SO_3_ also has excellent thermal stability (Fig. 1c).

Next, the adhesive force of inorganics was calculated, including the binding energies between the LCO surface (Fig. S3) and CEI components, and interfacial binding energies for the interior CEI layer (Fig. 1d and Fig. S4). It is clear that Li_2_SO_3_ exhibits the highest adhesive force, serving as a binding agent in the CEI layer and contributing to prevent the CEI from dissolution. Furthermore, the interaction force between heterogeneous inorganics would stretch the Li-anion bonds [20] and reduce the transport energy barrier of Li^+^, thus further facilitating fast Li^+^ conduction within the CEI (Fig. S5). Therefore, we propose a multi-inorganic strategy for CEI design under harsh conditions, comprising Li_3_PO_4_, LiF and Li_2_SO_3_. In this structure, Li_2_SO_3_ provides vital adhesiveness and works synergistically with Li_3_PO_4_ to enhance the thermal stability and ionic conductivity of the CEI layer, while LiF serves as a chemically stable and electron-blocking framework.

**Figure 2.**
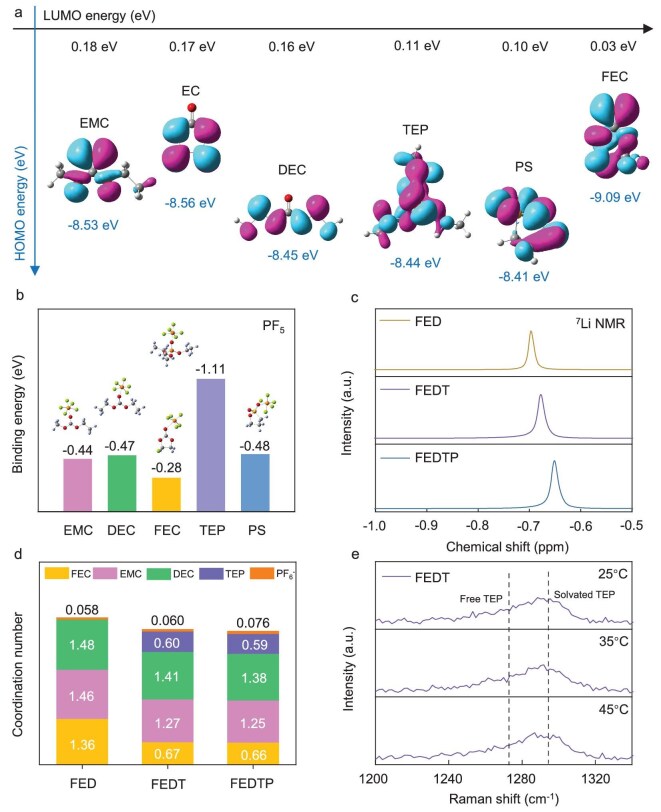
(a) Diagram of calculated HOMO/LUMO energies of electrolyte molecules; (b) binding energies of electrolyte molecules with PF_5_; (c) ^7^Li NMR spectra of various electrolytes at 25°C; (d) coordination environment of Li^+^ in various electrolytes at 45°C according to the molecular dynamics simulation; (e) Raman spectra of FEDT electrolyte at various temperatures.

The designed electrolyte (denoted as FEDTP) is composed of 1.0 M LiPF_6_ in fluoroethylene carbonate (FEC), ethyl methyl carbonate (EMC), diethyl carbonate (DEC) and TEP with a volume ratio of 2 : 4 : 3 : 1, which includes 1 vol% PS. For comparison, the electrolyte without adding PS (denoted as FEDT), the electrolyte without adding PS and TEP (denoted as FED) and the baseline carbonate electrolyte (denoted as EED, more detailed information can be found in the Supporting Information) were also prepared. At 45°C, the LCO cathode suffered a dramatic capacity loss in EED due to the unstable electrolyte and radical interface reaction (Fig. S6). In this view, EC solvent is replaced with FEC, which exhibits better oxidative stability. The linear sweep voltammetry (LSV) curves show that the FEDTP system shows an early oxidation peak, corresponding to the formation of the CEI (Fig. S7), which is consistent with the theoretical results (Fig. 2a). Besides, it has been reported that the autocatalytic decomposition of LiPF_6_ under elevated temperature leads to the formation of PF_5_, which would react with trace amounts water to form HF, resulting in severe damage of the CEI and battery degradation. The calculated binding energies (Fig. 2b) indicate that TEP could stabilize PF_5_ to inhibit HF formation, which is beneficial for the electrochemical cycling of the LCO cathode at elevated temperature [21]. To verify this speculation, the elevated temperature storage property of various electrolytes was tested (Fig. S8) and FED shows significant degradation due to HF formation.

The Li^+^ solvation structure was studied by Fourier transform infrared spectroscopy (FTIR, Fig. S9). The convoluted peaks around 1680–1880 cm^−1^ correspond to C=O stretching vibration of various solvents [22,23]. Quantitative analysis revealed the weakened coordination between the solvent and Li^+^ upon the introduction of TEP and PS. Considering the potential detection limit of FTIR for trace additive effect, nuclear magnetic resonance (NMR) spectra were applied, which more clearly reflect changes in the local coordination environment. The downfield chemical shift of ^7^Li observed in the NMR spectra (Fig. 2c) indicates a weaker shielding effect around Li^+^, hence implying the lowest Li^+^ de-solvation for the FEDTP electrolyte. Next, the coordination state was investigated by Raman spectroscopy (Fig. S10). The increased relative intensity of solvated PF_6_^−^ Raman peaks in both FEDT and FEDTP electrolytes suggests the formation of more CIPs [24]. With the gradual addition of TEP, a progressively increasing intensity of the Raman peak corresponding to solvated PF_6_^−^ was observed, indicating that more PF_6_^−^ ions coordinated with Li^+^ (Fig. S11). Together, the introduction of TEP solvent and PS additive effectively reduce the solvated number of carbonates so that PF_6_^−^ anions tend to participate in the solvation shell. Furthermore, the addition of TEP solvent and PS additive further improves the ionic conductivity of the electrolyte, thereby facilitating faster Li^+^ transport (Fig. S12).

In order to elucidate the effect of TEP and PS on the atom-level solvation sheath of Li^+^, molecular dynamics (MD) simulation and density functional theory (DFT) calculations were carried out (Figs S13–S15). In the FED electrolyte, EMC is the dominant solvent in the first Li^+^ solvation shell at 25°C. With the addition of TEP solvent, the coordination numbers of original carbonates drop sharply due to the higher binding energy with Li^+^ and the larger van der Waals volume of TEP molecules (Figs S16 and S17). Based on the statistical results in solvation shells, the average de-solvation energy of various electrolytes could be calculated using weighted average methods (Fig. S18) [25]. It could be clearly observed that the Li^+^ de-solvation energy barrier decreased after the introduction of TEP, further substantiating the above spectroscopic results where more Li^+^-PF_6_^−^ CIPs were formed. Besides, the remarkable increase of CIPs is found in the FEDTP system which could be largely attributed to the high affinity between PS and PF_6_^−^ (Fig. S19). When PS locates in the inner solvation sheath of Li⁺ (Fig. S13), it will induce more PF_6_^−^ close to Li^+^.

The temperature dependance of the Li^+^ solvation structure also significantly influences the electrochemical performance at elevated temperatures. Specifically, as the temperature increases from 25 to 45°C, intensified molecular thermal motion leads to a shift in solvation behavior: linear carbonates with higher binding energy (i.e. DEC) tend to enter the first solvation shell, whereas those with lower binding energy (i.e. EMC) exhibit a decrease in coordination number (Fig. 2d and Fig. S15). In contrast, TEP maintains a constant coordination number with Li^+^ at both temperatures, demonstrating low temperature sensitivity, which is an advantageous property for systems subjected to thermal variations. As shown in Fig. 2e, the vibration of P=O from free TEP is located at ∼1270 cm^−1^ and solvated peak is located at ∼1290 cm^−1^ [26,27]. With the rise in temperature, the characteristic peaks of TEP remained essentially unchanged, confirming the results of MD simulation.

### Electrochemical performance under HV and elevated temperature

**Figure 3.**
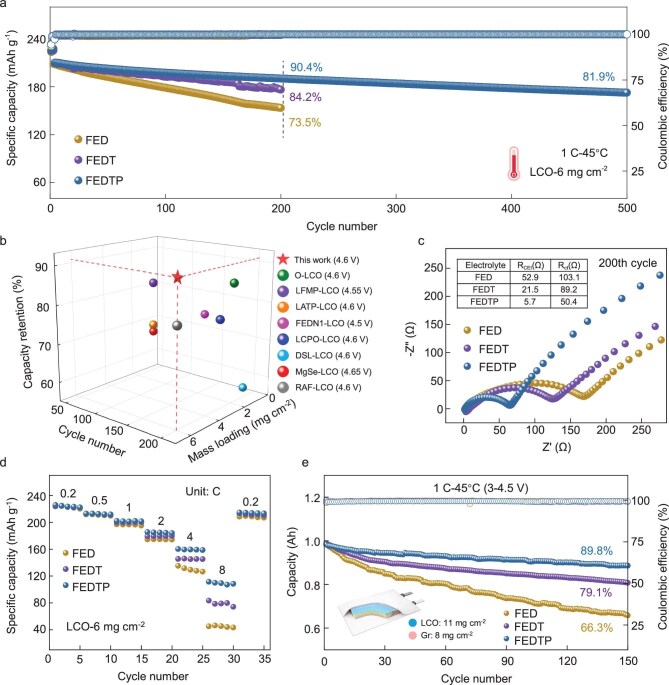
(a) Galvanostatic cycling performance and coulombic efficiency of LCO cathodes with various electrolytes at a rate of 0.2 C for the first 3 cycles and 1 C for subsequent cycles; (b) comparison of the LCO electrochemical performance under elevated temperature condition; (c) EIS of LCO cathodes with various electrolytes after 200 cycles; (d) rate performance of LCO cathodes with various electrolytes; (e) galvanostatic cycling performance and coulombic efficiency of pouch cells at a rate of 1 C with various electrolytes.

To highlight the significance of a compatible electrolyte system under harsh conditions, the electrochemical performance of various electrolytes was examined in the voltage range of 3.0–4.6 V (vs Li^+^/Li) at 45°C. As shown in Fig. 3a, the FEDTP system enables the highest specific capacity of 190 mAh g^−1^ (corresponding to 90.4% capacity retention) than that cycled with other electrolytes after 200 cycles at 1 C, with an enhanced average coulombic efficiency (CE) of 99.86%. Even after 500 cycles, the battery still retains an 81.9% capacity retention rate. By comparing the capacity retention and mass loading delivered by FEDTP-LCO cells with those recently reported LCO batteries under HV and elevated temperatures (Fig. 3b; see details listed in Table S1), this work exhibits state-of-the-art overall performance. In sharp contrast, the overpotentials of LCO electrodes rapidly increased after cycling in FED or FEDT (Fig. S20), inferring gradual interface deterioration and structural degradation of cathode materials. The cycling stability of LCO with the FEDP electrolyte (FED electrolyte with 1 vol% PS) was also evaluated, showing an improved capacity retention rate compared with the FED system (Fig. S21). The evolution of interfacial structures is confirmed by the Nyquist plots of half-cells after 200 cycles (Fig. 3c). The fitting results show that FEDTP displays significantly lower CEI impedance (*R*_CEI_) as well as transfer resistance (*R*_ct_) than other electrolytes. Combining the *in-situ* EIS of LCO cathodes during the 1st cycle (Fig. S22), the *R*_CEI_ and *R*_ct_ remained stable under lithiation in the FEDTP electrolyte, indicating the formation of a robust interphase with fast channels for Li^+^.

Apart from the cycle life, FEDTP also facilitates superior rate capability over FED and FEDT (Fig. 3d and Fig. S23), with a capacity of 110 mAh g^−1^ at 8 C. To explore the origin of improved cycle stability, the diffusion coefficient of Li^+^ (*D*_Li_) in the LCO cathode was calculated by the Randles–Ševčík equation according to the results of stepped sweep rate cyclic voltammetry (CV) (Fig. S24) [28]. The *D*_Li_ values of lithiation and delithiation of LCO in FEDTP are measured to be 4.11 × 10^−7^ and 2.69 × 10^−7^ cm^2^ s^−1^, respectively, about double those measured in FED (1.99 × 10^−7^ and 1.27 × 10^−7^ cm^2^ s^−1^). This finding well agrees with the 1st cycle CV curves of various electrolytes (Fig. S25), where FEDTP exhibits the narrowest and most intense reversible redox peaks, suggesting faster charge transfer kinetics. To investigate the kinetics of Li^+^ transfer across various CEIs, the activation energies of CEIs were calculated based on EIS at variable temperatures (Figs S26 and S27). Exhibiting the lowest activation energy (16.54 kJ mol^−1^), the CEI derived from FEDTP exhibits the lowest energy barrier for Li^+^ migration.

To further demonstrate the feasibility of our electrolyte design strategy under practical testing conditions, proof-of-concept LCO||graphite pouch cells (∼1 Ah) were assembled and tested between 3.0 and 4.5 V under 45°C (Fig. 3e). The cell cycled with FEDTP exhibited stable voltage profiles and voltage plateaus during cycling (Fig. S28). After 150 cycles, FEDTP delivered suppressed rates of capacity fading (ΔQ_FEDTP_ = 0.1 Ah) as well as voltage decay (ΔV_FEDTP_ = 0.02 V) than FEDT (ΔQ_FEDT_ = 0.18 Ah, ΔV_FEDT_ = 0.04 V) and FED (ΔQ_FED_ = 0.33 Ah, ΔV_FED_ = 0.12 V), demonstrating huge potential for practical applications. To exclude the potential impact of electrolytes on graphite negative electrodes, the electrochemical performance of Gr||Li half cells (Fig. S29) were further researched and there was no significant difference between different electrolytes. It has been reported that the significant thermal degradation of the SEI layer and graphite anode starts at temperatures >50°C [29,30]. Therefore, the electrochemical performance of full cells at 45°C was mainly dictated by the LCO cathode.

### Structural stability of LiCoO_2_

**Figure 4.**
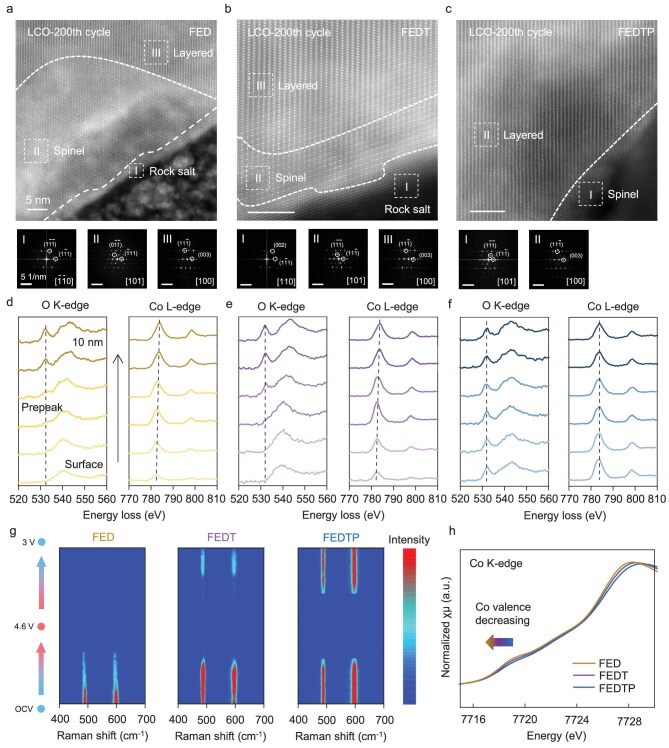
HAADF-STEM images of LCO cathodes with FED (a), FEDT (b) and FEDTP (c) electrolytes after 200 cycles at 45°C; fast Fourier transform (FFT) diffraction fringes corresponding to the marked areas in each TEM image are shown below the respective images; EELS spectra in the surface region of LCO cathodes with FED (d), FEDT (e) and FEDTP (f) electrolytes after 200 cycles at 45°C; (g) *in-situ* Raman spectra of LCO cathodes with various electrolytes in the first cycle at 45°C; (h) Co K-edge XANES spectra of LCO cathodes after 200 cycles at 45°C with various electrolytes.

Generally, the structural instability of cathode materials originates from the surface. Herein, high angle annular dark field-scanning transmission electron microscopy (HAADF-STEM) and corresponding electron energy loss spectroscopy (EELS) measurements were performed to investigate the near-surface phase structure and valence variations, respectively (Fig. 4a–f). For LCO cycled in FED, a substantial near-surface region of spinel phase can be observed, capped by an outermost rock-salt (RS) phase ∼5 nm thick. The above results suggest LCO underwent severe structural degradation in FED primarily due to unrestricted surface side reaction. In contrast, LCO cycled in FEDT exhibited significantly milder degradation, with a much thinner RS phase (∼2 nm) and a spinel phase (∼5 nm) extending from the surface toward the bulk (Fig. 4b). In stark contrast, the LCO maintains structural stability with minimal structural degradation (<2 nm spinel phase on the surface) after 200 cycles in FEDTP (Fig. 4c).

The near-surface structure of the cycled LCO is further identified by EELS (Fig. S30). The crystal field splitting of the Co-O octahedral configuration results in the generation of *t_2__g_* and *e_g_* bands, corresponding to peak I at ∼531.6 eV and bulge II at ∼544 eV, respectively [31]. For LCO cycled in FED, the disappearance of pre-peak in O K-edge spectra at ∼531.6 eV suggests the formation of lattice oxygen defects in the near-surface region due to severe side reactions (Fig. 4d). Besides, the shift (∼1.4 eV) of Co L_3_-edge peak to lower energy loss indicates that the surface Co^3+^ is reduced to Co^(3-x)+^ and mixed phases (e.g. CoO and Co_3_O_4_) are formed, accompanying Li^+^ deficiency to accelerate structural degradation. For FEDT samples, the absence of a pre-peak only exists in a limited area with a depth of 6 nm and a smaller shift (∼1 eV) of Co L_3_-edge peak is observed (Fig. 4e), suggesting a less damaged surface structure. In comparison, for the LCO cycled in FEDTP, stable pre-peaks are detected in O K-edge spectra, while the location and intensity of Co L-edge peaks remain unchanged in the whole detected area (Fig. 4f). It can be inferred from above results that the structural instability of LCO at 45°C originates and propagates from the surface toward the bulk.

To reveal the evolution of surface TM-O bond vibration on the LCO cathode during cycling, *in-situ* Raman spectra measurements were conducted (Fig. 4g) for the 1st cycle [32]. The vibration peaks at ∼500 and ∼600 cm^−1^ correspond to the O-Co-O bending vibrations (E_g_) and Co-O symmetrical stretching (A_1g_), respectively. During the reduction process, the intensity of vibration peaks attenuated significantly for LCO cathodes in FED and FEDT, suggesting irreversible lithiation progress and destabilization of Co-O bonds. By contrast, the Raman spectra of the FEDTP group exhibit excellent reversibility of O-Co-O and Co-O bonds. Next, *in-situ* electrochemical mass spectrometry (DEMS) experiments were also conducted to analyze the evolution of CO_2_ and O_2_ in various electrolytes (Fig. S31) [33]. For the FED electrolyte, the cell began to release CO_2_ and O_2_ at ∼20% state of charge (SOC), corresponding to carbonate solvent decomposition and surficial lattice O loss. In sharp contrast, no obvious gas evolution was detected with the FEDTP electrolyte, suggesting that both carbonate solvent decomposition and oxygen loss were effectively suppressed. X-ray absorption near-edge structure spectra at the K-edges of Co were collected after 200 cycles (Fig. 4h). The absorption edge of Co for FEDTP shows a shift to higher energy, indicating higher average valence states of the Co element compared with that in FED and FEDT. This is consistent with the results of EELS and DEMS. Furthermore, the loss of active oxygen species is usually accompanied by dissolution of low-valence TM ions. Both XPS and inductively coupled plasma atomic emission spectroscopy (ICP-AES) results of the cycled Li anode surface with different electrolytes confirmed a large amount of Co^2+^ dissolved and shuttled to Li in FED (Figs S32 and S33), which can be mitigated in both FEDT and FEDTP. Besides, the lattice parameters *a* and *c* calculated by XRD data for the LCO cathode cycled in the FEDTP electrolyte deviate less from pristine LCO than those in other electrolytes (Figs S34 and S35), indicating the improved reversibility of the bulk structure. From above results, it can be concluded that our electrolyte design strategy stabilizes the crystal structure of LCO at the (near-)surface by suppressing lattice O loss and Co dissolution as well as violent decomposition of the electrolyte.

### Constructing robust cathode electrolyte interphase

**Figure 5.**
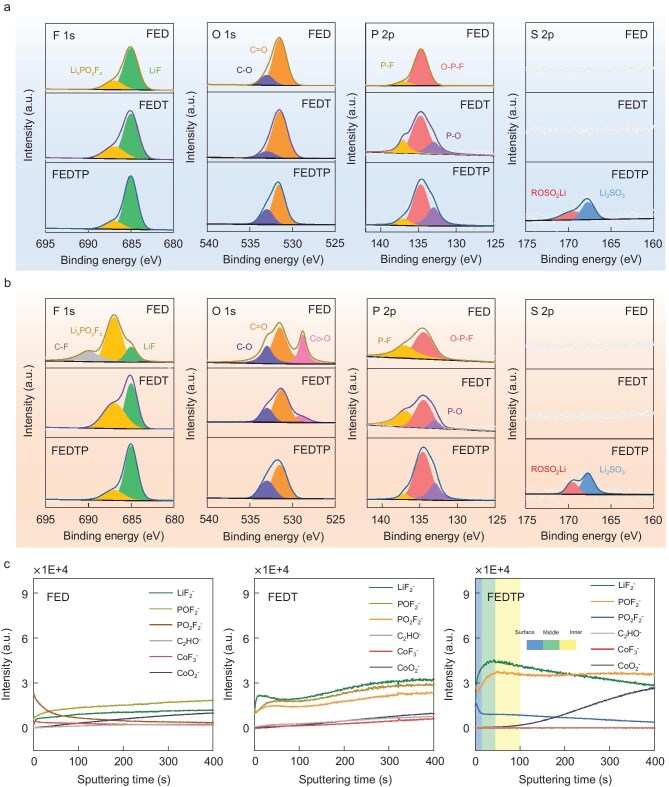
F 1s, O 1s, P 2p and S 2p X-ray photoelectron spectroscopy (XPS) spectra of LCO cathodes with various electrolytes after 200 cycles at 25°C (a) or 45°C (b); (c) depth profiles of various secondary ion fragments during the ToF-SIMS measurement for LCO cathodes with various electrolytes after 200 cycles at 45°C.

Different from the results obtained at 45°C, galvanostatic cycling of LCO cathodes with FED, FEDT and FEDTP electrolytes at 25°C exhibited very similar capacity retention rates (Fig. S36). Next, X-ray photoelectron spectroscopy (XPS) was employed to reveal the compositional evolution of CEI. At 25°C, CEI components formed in all three electrolytes are very similar, except that peaks at ∼133 eV and ∼168 eV can be observed after the introduction of TEP and PS, respectively, suggesting the formation of Li_3_PO_4_ and Li_2_SO_3_ (Fig. 5a). This result agrees well with the cycling performance of the three electrolytes at room temperature.

However, at 45°C, the chemical composition of the CEI layer in FED changed dramatically (Fig. 5b), while the CEI evolution in FEDTP barely changed under operation at elevated temperature. The F 1s spectra show that the high LiF content in FEDTP is well preserved, without showing the increasing amount of undesirable decomposition species of LiPF_6_ (e.g. Li_x_PF_z_, Li_x_PO_y_F_z_) in the other two electrolytes, especially FED. The appearance of C-F bonds (corresponding to the polyvinylidene fluoride binder) also indicates that the CEI formed on LCO in FED completely fails at 45°C, resulting in a large amount of exposed electrode surface. This result is well corroborated by the O 1s spectra, where a significant Co-O peak (∼529 eV) could be observed in FED, indicating exposed LCO particles. When compared with FED, FEDT effectively reduced the Co-O peak whereas the FEDTP-derived CEI managed to protect the cathode after 200 cycles at 45°C. From the P 2p and S 2p spectra, it can be observed that both Li_3_PO_4_ and Li_2_SO_3_ components remain stable at 200 cycles at 45°C, well agreeing with calculation results. The CEI components formed in the initial cycling stage (3 cycles) of various electrolytes at 45°C were also examined by XPS (Fig. S37). The initial CEI formed by all electrolytes at 45°C exhibits a composition similar to that shown in Fig. 5a, indicating that the CEI structure remains relatively intact at this stage. However, during prolonged cycling, the CEI generated in FED degraded rapidly, whereas the stepwise introduction of TEP and PS sequentially facilitated the formation of heat-tolerant Li_3_PO_4_ and adhesive Li_2_SO_3_, thereby ensuring that the CEI maintains its morphological and compositional stability at elevated temperatures.

Furthermore, *in-situ* FTIR was performed to reveal the interfacial evolution of various electrolytes during CEI formation (see the schematic setup in Fig. S38). Surface-enhanced *in-situ* FTIR spectra (Fig. S39) show that during the charge process from OCV to 4.6 V, the stepwise addition of TEP and PS significantly inhibited carbonate decomposition, as evidenced by the diminished reverse peak intensities of FEC (∼1830 cm^−1^), Li^+^-FEC (∼1800 cm^−1^), EMC/DEC (∼1742 cm^−1^) and Li⁺-EMC/DEC (∼1708 cm^−1^) peaks. Subsequently, the oxidation pathways of TEP and PS were further investigated via DFT calculations (Fig. S40). Upon electron loss, both TEP and PS exhibit a tendency to cleave the C-O bond. Based on the above experimental results and theoretical analysis, detailed decomposition pathways of TEP and PS are proposed (Fig. S41). The decomposition of TEP leads to the formation of Li_3_PO_4_ and ethylene, while PS decomposes into Li_2_SO_3_ and organosulfite species such as RSO_3_Li. Given their structural similarity to Li_2_SO_3_, the minor organosulfite species (RSO_3_Li) are reasonably expected to possess adhesion characteristics. The detection of ethylene signal in DEMS test and Li_3_PO_4_ signal in XPS test (using LiClO_4_ as Li-salt) further confirms the decomposition path of TEP (Fig. S42).

To reveal the spatial distribution of various chemical components within the CEI, time-of-flight secondary-ion mass spectrometry (ToF-SIMS) was performed on cycled LCO electrodes (Fig. S43 and Fig. 5c). For FEDTP, it could be observed that after long cycling, the cathode is covered by a stable CEI composed of LiF_2_^−^, PO_3_^−^ and SO_3_^−^ fragments. In addition, the S 2p XPS results after etching (Fig. S44) show that Li_2_SO_3_ is primarily located in the outermost layer of the CEI. By combining XPS with ToF-SIMS results, it is concluded that Li_3_PO_4_, LiF and Li_2_SO_3_ formed in FEDTP are predominantly distributed in the inner, middle, and outer regions of the CEI, respectively [34,35]. This trilayered CEI structure aligns well with the design concept illustrated in Fig. 1, where the inner Li_3_PO_4_ stabilizes the surficial oxygen of the LCO cathode, while the outer Li_2_SO_3_ firmly adheres to LCO and other CEI inorganics, forming a compact protective cap that effectively suppresses the dissolution of the entire CEI layer.

Furthermore, the significantly weaker intensity of C_2_HO^−^ in FEDTP demonstrated the suppressed decomposition of carbonate solvent. In contrast, a large number of POF_2_^−^ and PO_2_F_2_^−^ fragments were detected on the LCO surface in FED, suggesting the excessive decomposition of LiPF_6_. The broken distribution of LiF_2_^−^ was deduced to relate to the instability of CEI under 45°C that causes dissolution of organic species, hence the detachment of LiF. After the addition of TEP, more distinctive LiF_2_^−^ fragments could be observed on the LCO surface, accompanied by PO_2_^−^ and PO_3_^−^ species, indicating partial dissolution of the CEI layer. However, the widespread presence of carbonate decomposition products (C_2_HO^−^ species) still indicates that the interface of LCO is not effectively passivated. Besides, the CoF_3_^−^ fragments are the dissolved products of Co attacked by electrolyte, while the CoO_2_^−^ fragments are considered as an indication of LCO cathode materials. Due to the effective retention of CoO_2_^−^ components (Fig. S45) and the minimal presence of CoF_3_^−^ components, this result further confirms that FEDTP can effectively suppress side reactions between the electrolyte and LCO [36,37].

**Figure 6.**
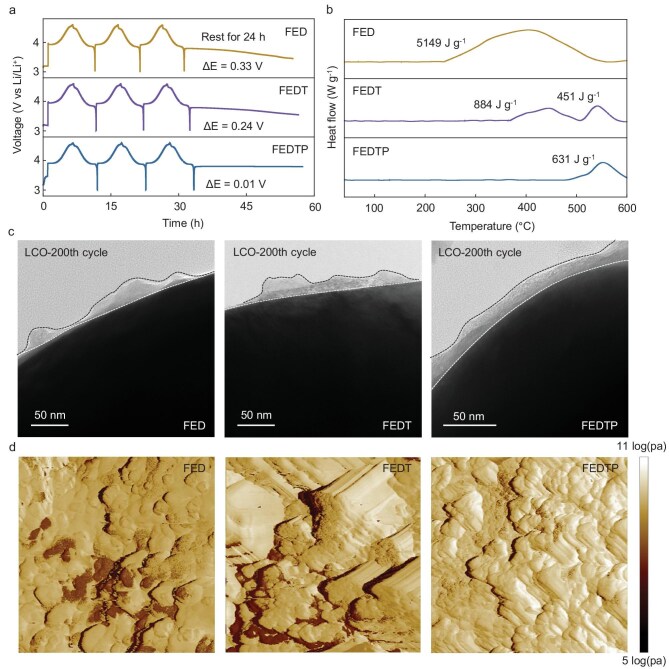
(a) Time-voltage curves of LCO cathodes with various electrolytes under 0.2 C current density at 45°C; (b) DSC curves of LCO cathodes under lithiation state with various electrolytes after 200 cycles at 45°C; (c) cryo-TEM images of LCO cathodes with FED, FEDT and FEDTP electrolytes after 200 cycles at 45°C; (d) mechanical property of CEI on the LCO cathodes with FED, FEDT and FEDTP electrolytes after 200 cycles at 45°C measured *via* AFM (2 μm × 2 μm).

The thermal stability of the CEI layer was further investigated through electrochemical characterization and differential scanning calorimetry (DSC) measurement. At 45°C, by allowing the half-charged cells to rest for 24 h, the cells with FED and FEDT electrolytes exhibited noticeable potential drops, indicating continuous dissolution of the CEI (Fig. 6a). In sharp contrast, the cell with FEDTP exhibited minimal potential drop, showing that the CEI remains stable and is not readily dissolved, even under prolonged elevated temperature storage. A floating charge test was also conducted to evaluate the side reactions between the fully charged cathode and electrolyte (Fig. S46). Compared with the FED and FEDT electrolytes, LCO cycling in the FEDTP electrolyte exhibited significantly lower leakage currents throughout the entire floating period (100 h), indicating reduced parasitic reactions and enhanced electrochemical stability. As shown in Fig. 6b, the total heat-releasing of LCO cycling in the FEDTP electrolyte (∼631 J g^−1^) is lowest compared with that of LCO cycling in the FED electrolyte (∼5149 J g^−1^) and FEDT electrolyte (∼1335 J g^−1^), suggesting that the most compact and inorganics-rich CEI layer formed in the FEDTP electrolyte. Moreover, the decomposition temperature of the CEI formed in FEDTP was delayed to ∼483°C, indicating enhanced thermal stability of the CEI layer.

Next, the CEI's microstructure formed in different electrolytes was studied by cryo-transmission electron microscopy (cryo-TEM). After cycling in the FED electrolyte at HV and 45°C, a broken and thick surface layer was observed on the LCO cathode which cannot provide effective protection (Fig. 6c). In sharp contrast, the use of TEP solvent and PS additive results in a thin and uniform CEI layer that passivates the LCO surface without compromising Li^+^ transfer. Combined with scanning electron microscopy (SEM) results, obvious dissolution of the CEI was found in the FED and FEDT electrolyte (Fig. S47), exposing the highly active Co on the cathode surface and inducing repeated electrolyte consumption. Then, atomic force microscopy (AFM) was applied to characterize the roughness and mechanical properties of CEI layers on the LCO surface with various electrolytes (Fig. 6d,  Figs S48 and S49). The LCO particle cycled in FEDTP shows a more uniform and smoother surface morphology compared to that cycled in the FED and FEDT electrolytes, which is consistent with the results of SEM and cryo-TEM. After logarithm transformation of the Derjaguin-Muller-Toporov (DMT) modulus, the CEI formed in FEDTP exhibits a more uniform distribution with a higher average value (9.74 Log(Pa)) compared with those formed in FED (9.25 Log(Pa)) and FEDT (9.35 Log(Pa)), manifesting a denser inorganics-rich CEI layer. In contrast, the CEI layer rich in organic components with damaged structures naturally displays poorer mechanical properties.

### Revealing the improving mechanism of LiCoO_2_

As shown in Fig. S50, the CEI formed in the FED system primarily consists of LiF and RPO_2_F_2_, which can disintegrate (e.g. through decomposition and dissolution in the electrolyte) under elevated temperature and high-voltage conditions, resulting in an incoherent distribution. To address the limitations of a single inorganic component, two additional inorganic species with excellent thermal stability (Li_3_PO_4_ and Li_2_SO_3_) were introduced into the CEI. On one hand, the addition of TEP helps inhibit HF formation and generates inorganic Li_3_PO_4_, which stabilizes the interfacial lattice oxygen of the LCO cathode *via* PO_4_^3−^ and accelerates Li⁺ transport across the CEI. On the other hand, the strong adhesion of Li_2_SO_3_ effectively anchors other CEI components to the LCO surface. The combination of these three components and their spatial distribution achieves a breakthrough in the densification of the CEI, which effectively passivates the high-activity sites on the cathode surface under high-voltage conditions and prevents the dissolution of organic materials at elevated temperatures. Thus, the heterogeneous inorganics comprising the dense CEI enhance interfacial stability at elevated temperatures and improve the electrochemical performance of the HV-LCO cathode.

## CONCLUSION

In summary, through introducing TEP and PS into the electrolyte, a trilayered CEI, comprising Li_3_PO_4_, LiF and Li_2_SO_3_, which arranged hierarchically from the innermost to the outermost layer, is constructed for a high-voltage cathode at elevated temperature. We systematically integrated the synergistic effects of multiple inorganic CEI components through well-established additives. The electrochemically tailored interphase rich in LiF, Li_3_PO_4_ and Li_2_SO_3_ inorganic components not only restricts the excessive oxidation decomposition of the electrolyte, but also passivates the Co^2+^ catalytic sites and restrains the TM dissolution. The designed CEI exhibits favorable thermal stability and adhesion force which protects the structural integrity of the LCO cathode under severe conditions. Furthermore, the modified CEI layer markedly improves the charge transfer kinetics meaning that commercial pouch cells (1 Ah) could achieve impressive cycling performance. The proposed design strategy of the CEI greatly expands the application fields of LCO cathodes and is suitable for other cathode materials.

## Supplementary Material

nwaf345_Supplemental_File
