# Supplementary material for: Engineering durable interphases for high-voltage Li-ion batteries under thermal stress
Source: Natl Sci Rev. 2025 Aug 21;12(10):nwaf345. doi: 10.1093/nsr/nwaf345 (PMC12485966; doi:10.1093/nsr/nwaf345)
Supplement: nwaf345_Supplemental_File [file nwaf345_supplemental_file.pdf]

Supporting Information

Engineering Durable Interphases for High-voltage Li-ion Batteries under Thermal Stress

Shiming Chen[1,#], Wenguang Zhao[1,#], Guorui Zheng[2,*], Chenyu Yang[3], Taowen Chen[1], Hengyu Ren[1], Yue Zuo[1], Xiangming Yao[1], Ke Li[1], Haoyu Xue[1], Jianjun Fang[1], Yuxiang Huang[4], Kai Yang[5], Zu-Wei Yin[6], Luyi Yang[1,*] and Feng Pan[1,*]

[1]School of Advanced Materials, Peking University Shenzhen Graduate School, Shenzhen 518055, China;

[2]Institute of Materials Research (IMR), Tsinghua Shenzhen International Graduate School, Tsinghua University, Shenzhen 518055, China;

[3]National Synchrotron Radiation Laboratory, University of Science and Technology of China, Hefei 230029, China;

[4]Department of Chemistry, University of Hong Kong, Hong Kong 999077, China;

[5]Advanced Technology Institute, University of Surrey, Guildford GU2 7XH, UK;

[6]College of Energy, Xiamen University, Xiamen 361005, China

[#]Equally contributed to this work.

***Correspondence authors**. E-mails: zhengguorui1991@163.com; yangly@pkusz.edu.cn; panfeng@pkusz.edu.cn

1. Experimental Section

1.1 Materials

N-methyl pyrrolidone (NMP) and polyvinylidene fluoride (PVDF) were purchased from Aladdin. Micro-sized $LiCoO_2$ (LCO) was synthesized according to the previous work.[1] Graphite was kindly provided by Shenzhen BTR New Material Group Co., Ltd., . The electrolyte components (ethylene carbonate EC, fluoroethylene carbonate FEC, ethyl methyl carbonate EMC, diethyl carbonate DEC, triethyl phosphate TEP and 1,3-propane sultone PS) were kindly obtained from DoDoChem (Suzhou, China).

1.2 Electrochemical measurements

The cathodes were made by a typical slurry casting method with active material (LCO), conductive agent (acetylene black), and binder (PVDF) at a mass ratio of 9:0.5:0.5. The slurry was casted onto Al foil with a blade and dried in a vacuum at 100°C for 12 h. Then cathodes were cut into disks with a diameter of 10 mm which mass loading of active materials was about 6 mg cm^{-2}. The graphite anodes were made in the same method coated onto the Cu current collector with 1.8 mg cm^{-2} mass loading. The separator was porous PP films (Celgard 2500). The first baseline electrolyte is composed of 1.0 M $LiPF_6$ in 3:4:3 (v/v) EC/ EMC/DEC (denoted as EED). The second baseline electrolyte is composed of 1.0 M $LiPF_6$ in 3:4:3 (v/v) FEC/ EMC/DEC (denoted as FED). The third baseline electrolyte is composed of 1.0 M $LiPF_6$ in 2:4:3:1 (v/v) FEC/EMC/DEC/TEP (denoted as FEDT). The experimental group electrolyte is composed of 1.0 M $LiPF_6$ in 2:4:3:1 (v/v) FEC/EMC/DEC/TEP/ with 1 vol% PS (denoted as FEDTP). The galvanostatic cycling tests were carried out using a Neware battery test system within the voltage range of 3 V-4.6 V (vs. Li/Li^+) for LCO cathodes and 0.01 V-1 V(vs. Li/Li^+) for graphite anodes. The 2032-coin cells were charged and discharged at 0.2 C (1 C = 200 mAh g^{-1} for LCO, 1 C = 375 mAh g^{-1} for graphite) for initial three cycles. Cyclic voltammograms (CV) and electrochemical impedance spectroscopy (EIS) experiments were performed on Solartron Analytical 1470E electrochemical workstation. In-*situ* EIS measurements were under the current density of 0.2 C. The diffusion coefficient of Li^+ (D_{Li}) is calculated by the Randles-Sevcik equation: $I_p= (2.69 \times 10^5)\ AC_{Li}D_{Li}^{1/2}n^{3/2}v^{1/2}$.

1.3 Material characterizations

X-ray diffraction (XRD) patterns of LCO cathodes were collected by using Bruker D8 Advance diffractometer with a Cu-Kα radiation source (λ = 0.154 nm). The top-view morphology of cycling

cathodes was conducted by Field-emission scanning electron microscopy (SEM, ZEISS SUPRA55, Carl Zeiss). High-resolution field-emission transmission electron microscopy (FETEM) (JEOL-3200FS) was used to study the structure of cathodes and cathode electrolyte interphase (CEI) with cryo transfer tomography holder (model2550, Fischione). Electron energy loss spectroscopy (EELS) spectra of LCO cathodes are acquired from JEOL ARM 200F scanning transmission electron microscope (STEM) operated at 200 keV, equipped with a cold field emission gun and integrated aberration (Cs) corrector. ^{7}Li nuclear magnetic resonance (NMR) were recorded on a Quantum-I Plus 400 MHz NMR spectrometer at 298 K. The chemical states of selected elements in cathodes were analyzed by X-ray photoelectron spectra (XPS) (ESCALAB 250Xi). A time-of-flight secondary ion mass spectrum (TOF-SIMS) was measured on an ION-TOF GmbH TOF-SIMS 5-100 spectrometer. The typical sputtering area was 250 × 250 μm. In-*situ* Raman measurements were taken by Renishaw InVia Raman microscopy with a 633 nm laser, which irradiates the surface of cathode though Li metal foil and glass fiber separator with a 2 mm radius hole. In-*situ* FTIR measurements were conducted using a Nicolet iS50 FT-IR spectrometer (Thermo Fisher Scientific) equipped with a single-reflection diamond attenuated total reflection (ATR) sample cell. The instrument was fitted with a broad-band mercury-cadmium-telluride (MCT) detector, which was cooled using liquid nitrogen. For electrode preparation, the LCO slurry was cast onto separator and dried at 60°C for 12 hours. Then the LCO cathode was pressed tightly against the diamond window to serve as the working electrode, while a Li anode was placed on the other side of the separator as the counter electrode. A heating sheet is attached to the sample cell to maintain the system temperature at 45□ during the measurement. An Au film was produced via physical vapor deposition. The ICP-OES (HORIBA JY2000-2) was conducted for transition metal dissolution analysis. The surface morphology and mechanical property of CEI layer were measured by Bruker multimode 8 atomic force microscope under Ar atmosphere. Thermal stability tests of CEI were measured by TGA-DSC (PerkinElmer STA 6000) equipment in the Ar environment with 10 □ per minute heating rate.

1.4 Theoretical calculation

Density functional theory (DFT) calculations were based on the Gaussian 09 package. The B3PW91/6-311++G** theory level was used to optimize the molecular structure and perform frequency analysis to calculate HOMO/LUMO energy of molecule. Eps constant of 89.6 and epsinf constant of 1.9 were used for implicit solvation model (SMD) calculations.

Energy calculations were performed under theory level (B3LYP/6-311++G**) with Grimme's-d3 (gd3bj) dispersion correction to obtain the optimized coordination structures, calculating the binding energy under vacuum. The binding energy was calculated by the following equation: $E_{binding} = E_{totally} - E_x - E_y$. All energy calculation is corrected with the zero-point energy (ZPE).

Ab Initio Molecular Dynamics (AIMD) simulations were conducted through the Vienna Ab initio Simulation Package (VASP) to investigate the dynamic behavior of LiF, Li_2SO_3, and Li_3PO_4 crystals at a temperature of 318 K. The simulations were performed under the NVT ensemble using a Nosé-Hoover thermostat to accurately maintain the temperature throughout the simulation duration of 100 ps. The projector augmented wave (PAW) method was utilized for describing the ion-electron interactions, while the exchange-correlation energy was accounted for by the Perdew-Burke-Ernzerhof (PBE) functional within the generalized gradient approximation (GGA). To ensure computational efficiency without sacrificing accuracy, a minimal Γ-centered 1×1×1 k-point grid was chosen for the Brillouin zone sampling.

The calculation for density of states was studied by VASP software. A plane wave cutoff of 520 eV was applied for all structures. The convergence criterion for ionic relaxations was 10^{-6} eV per supercell and the Hellmann-Feynman force was converged to 0.01 eV □$^{-1}$. Spin polarization was taken into account and the Methfessel-Paxton method was employed to determine electron occupancies with a smearing width of 0.05 eV in all cases. The van der Waals-D3(Becke-Jonson) exchange-correlation functional was used for interface structures. This particular exchange-correlation functional is capable of capturing van der Waals interactions, which play an important role in surface interaction.

Molecular dynamics (MD) simulations were conducted by the Large Scale Atomic/Molecular Massively Parallel Simulator (LAMMPS). The simulation boxes were filled with 30 $LiPF_6$ + 123 FEC + 116 EMC + 74 DEC (FED), 30 $LiPF_6$ + 82 FEC + 116 EMC + 74 DEC + 18 TEP (FEDT), and 30 $LiPF_6$ + 82 FEC + 116 EMC + 74 DEC + 18 TEP + 3 PS (FEDTP). Atomic charges were calculated in Gaussian 09 using B3PW91/6-311++G** theory level. The systems were equilibrated at 298 K or 318 K in the isothermal-isobaric (NPT) ensemble for 5 ns using the Parrinello-Rahman barostat to maintain a pressure of 1 bar with a time constant of 1 ps. Finally, another 10 ns simulation in NVT ensemble under Nose-Hoover thermostats at 298 K or 318 K were performed.

2. Additional figures and tables

Table S1. Electrochemical performance comparison of recently reported high-voltage LCO.

	Voltage	Mass loading	Stability	Ref
FEDTP-LCO	4.6 V	6 mg cm^{-2}	90.8% capacity retention after 200 cycles at 1 C, 81.9% capacity retention after 500 cycles (45°C)	This work
O-LCO	4.6 V	2 mg cm^{-2}	86.1% capacity retention after 200 cycles at 1 C (45°C)	[2]
LFMP-LCO	4.55 V	3 mg cm^{-2}	84.1% capacity retention after 100 cycles at 1 C (55°C)	[3]
05MP-LCO	4.6 V	/	82% capacity retention after 200 cycles at 1 C (45°C)	[4]
LATP-LCO	4.6 V	3 mg cm^{-2}	72.9% capacity retention after 100 cycles at 0.5 C (45°C)	[5]
FEDN1-LCO	4.5 V	2.4 mg cm^{-2}	~75% capacity retention after 140 cycles at 0.5 A g^{-1} (30°C)	[6]
CM-LCO	4.6 V	/	76.6% capacity retention after 100 cycles at 1 C (50°C)	[7]
DPD-F-LCO	4.5 V	/	88.2% capacity retention after 200 cycles at 1 C (60°C)	[8]
LCPO-LCO	4.6 V	3 mg cm^{-2}	78% capacity retention after 200 cycles at 1 C (55°C)	[9]
DSL-LCO	4.6 V	3.5 mg cm^{-2}	57.6% capacity retention after 200 cycles at 1 C (45°C)	[10]
LiAlF-LCO	4.6 V	/	83.7% capacity retention after 100 cycles at 0.3 C (45°C)	[11]
MgSe-LCO	4.65 V	3 mg cm^{-2}	~71.1% capacity retention after 100 cycles at 1 C (45°C)	[12]
RAF-LCO	4.6 V	6 mg cm^{-2}	~80% capacity retention after 200 cycles at 0.3 C (45°C)	[13]
LCO-CAF	4.6 V	4 mg cm^{-2}	~75% capacity retention after 200 cycles at 1 C (45°C)	[14]
11N-LCO@LP	4.6 V	5 mg cm^{-2}	~71.9% capacity retention after 200 cycles at 1 C (45°C)	[15]
LCO-FPE	4.6 V	3 mg cm^{-2}	~76% capacity retention after 200 cycles at 1 C (45°C)	[16]

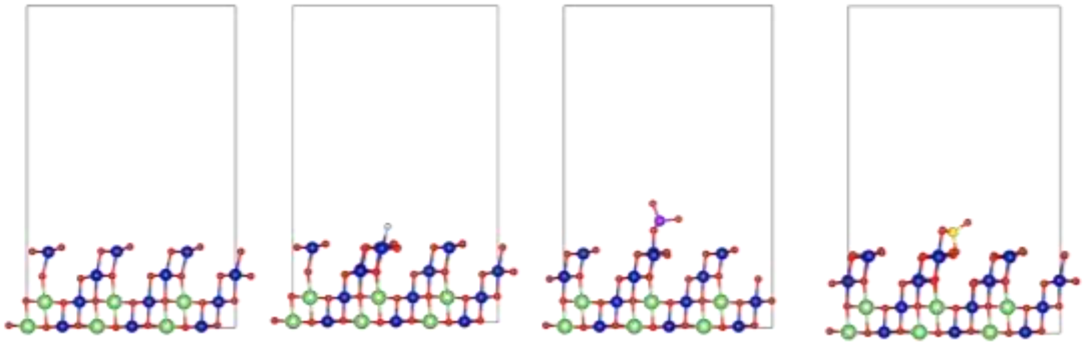

Figure S1. Optimized structure of various highly delithiated LCO (104).

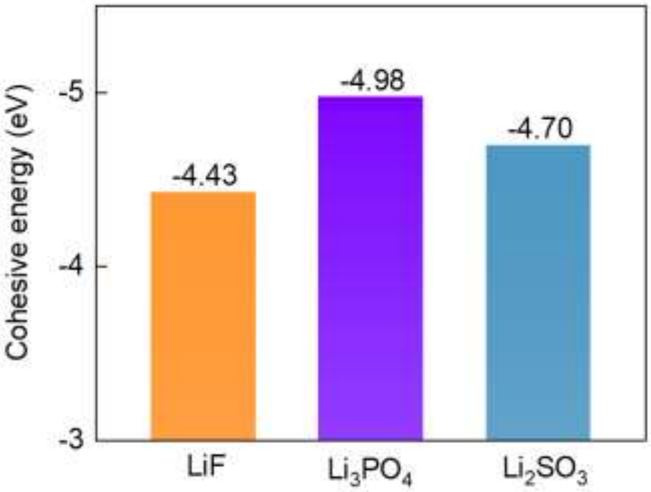

Figure S2. Cohesive energies of LiF, Li_3PO_4 and Li_2SO_3.

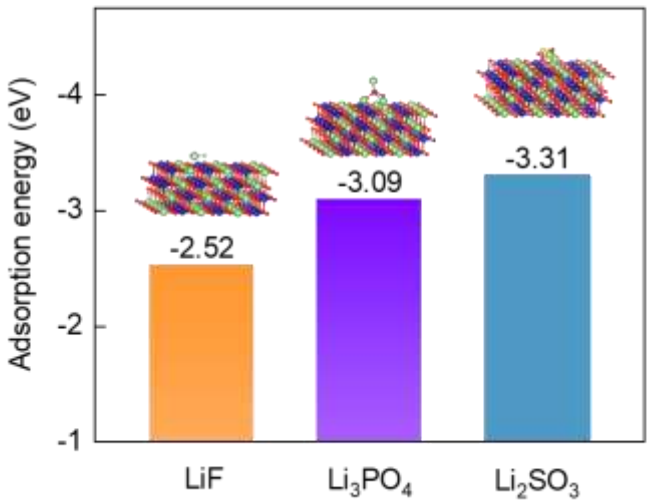

Figure S3. Adsorption energies between LCO surface (104) and LiF, Li_3PO_4 and Li_2SO_3.

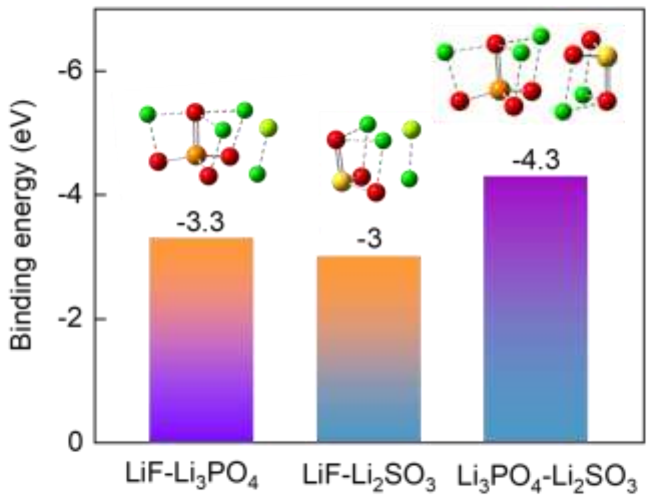

Figure S4. Binding energies between LiF, Li_3PO_4 and Li_2SO_3 molecules.

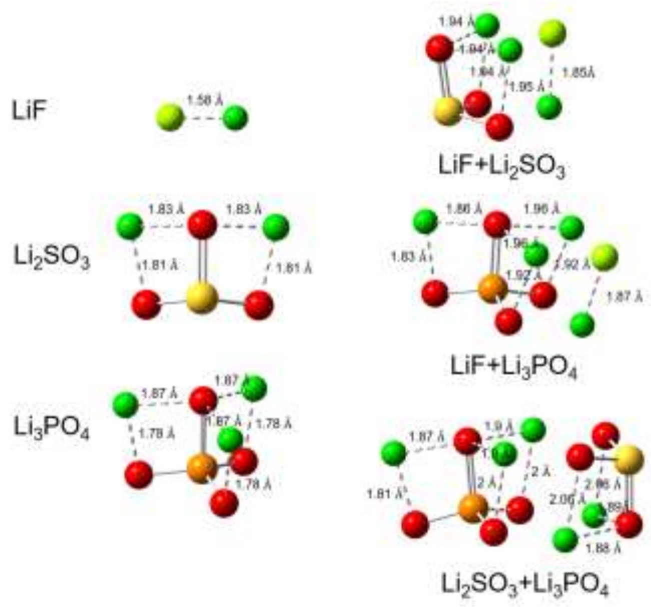

Figure S5. Optimized the structure of LiF, Li_3PO_4, Li_2SO_3, LiF-Li_3PO_4, LiF-Li_2SO_3 and Li_3PO_4-Li_2SO_3.

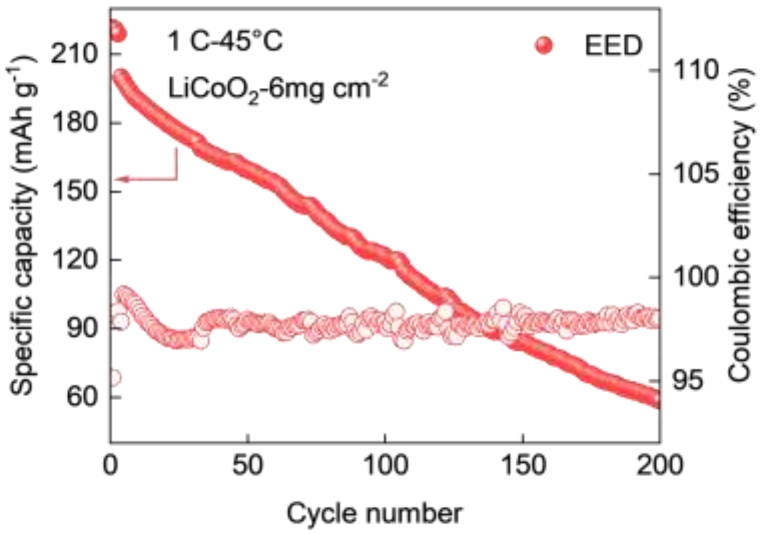

Figure S6. Galvanostatic cycling performance and coulombic efficiency of LCO cathodes with EED electrolyte at a rate of 0.2 C for the first 3 cycles and 1C for the subsequent cycles.

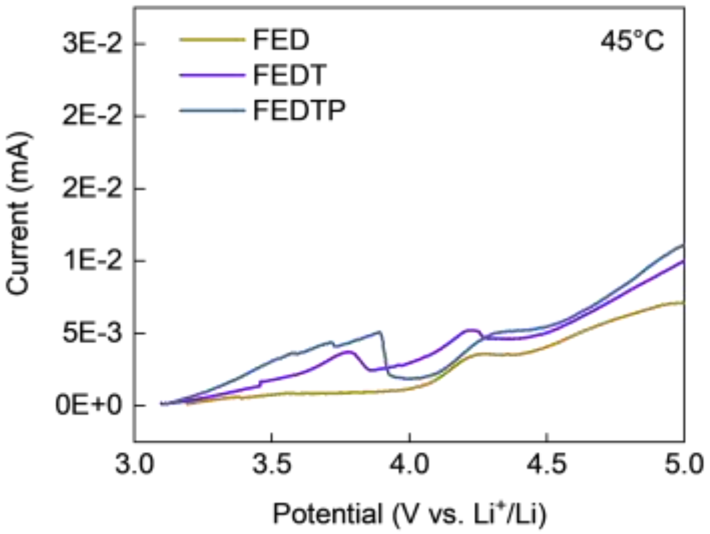

Figure S7. Comparison of LSV curves of various electrolyte: Li||steel positive sweep at 45°C.

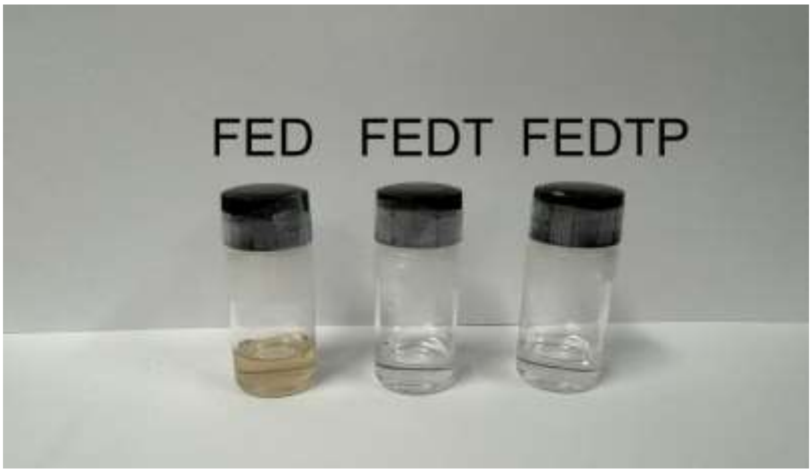

Figure S8. Optical photo of various electrolytes stored for one week at 60°C.

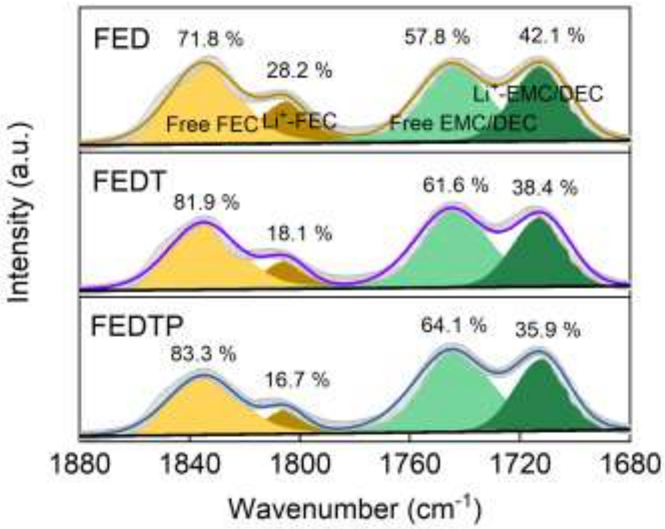

Figure S9. FTIR spectra of various electrolytes at 25°C.

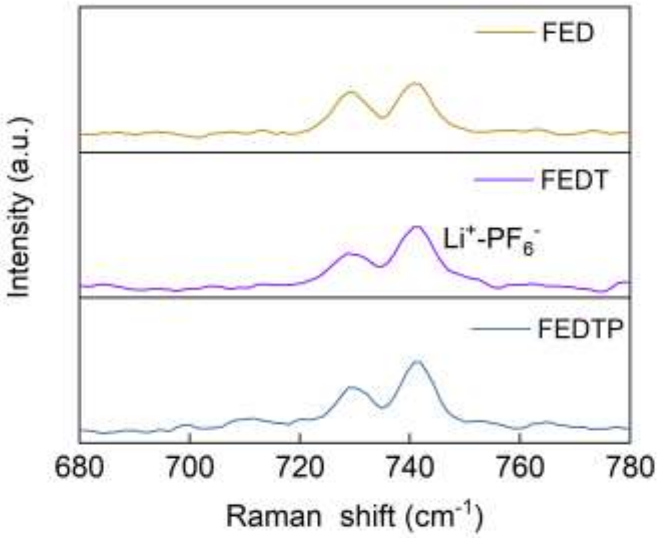

Figure S10. Raman spectra of various electrolytes at 25°C.

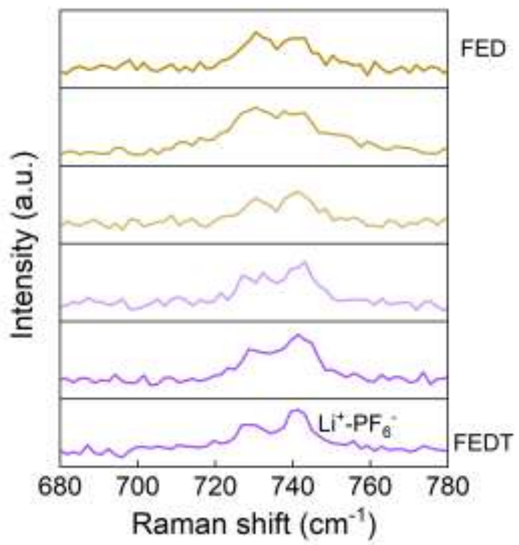

Figure S11. Raman spectra recorded during the addition of 1.0 M $LiPF_6$ in 1:4:3:2 (v/v) FEC/EMC/DEC/TEP solution into 0.5 mL of the FED electrolyte at 25°C, with 0.1 mL added per step.

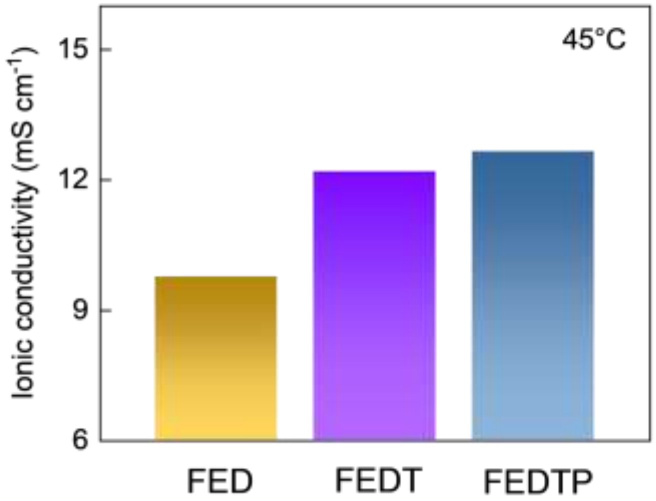

Figure S12. Ionic conductivity of various electrolytes at 45°C.

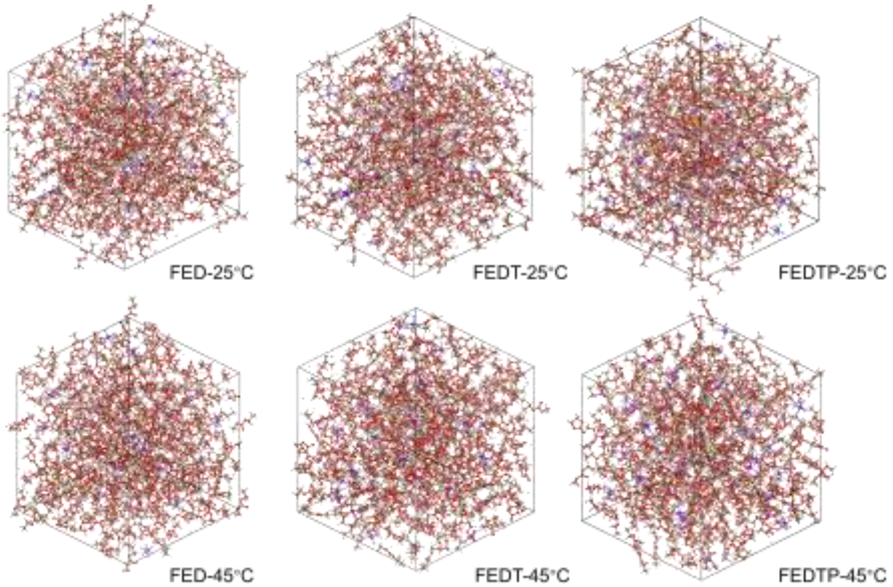

Figure S13. Snapshots of the MD simulation with various electrolytes at 25°C or 45°C.

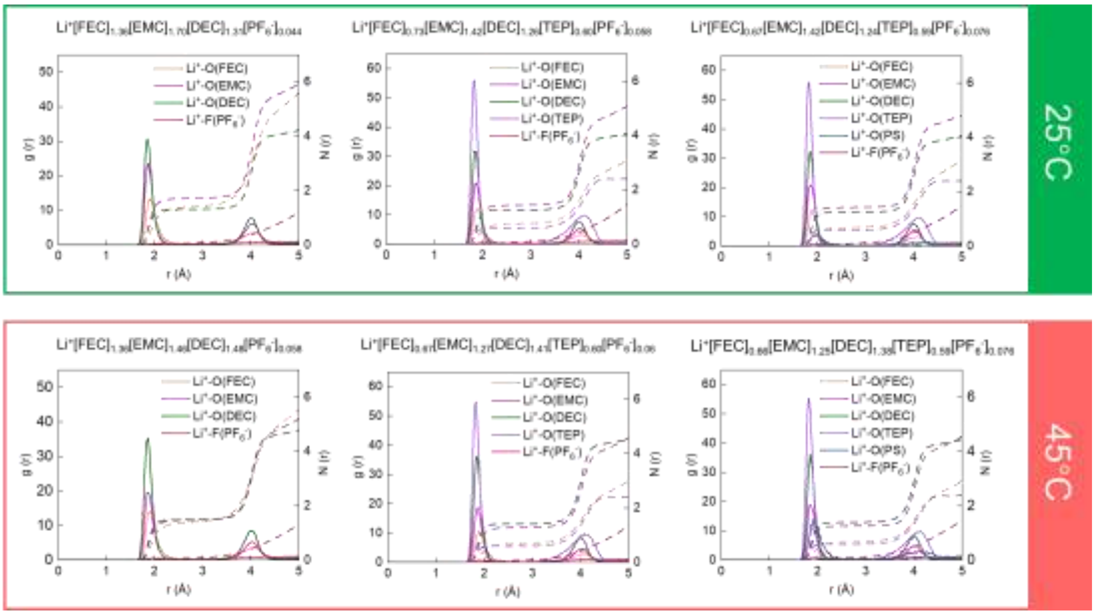

Figure S14. Radial distribution functions (RDF) and corresponding coordination numbers (N(r)) of various electrolytes at 25 □ or 45 □ (the first solvation radius is 2.51 Å).

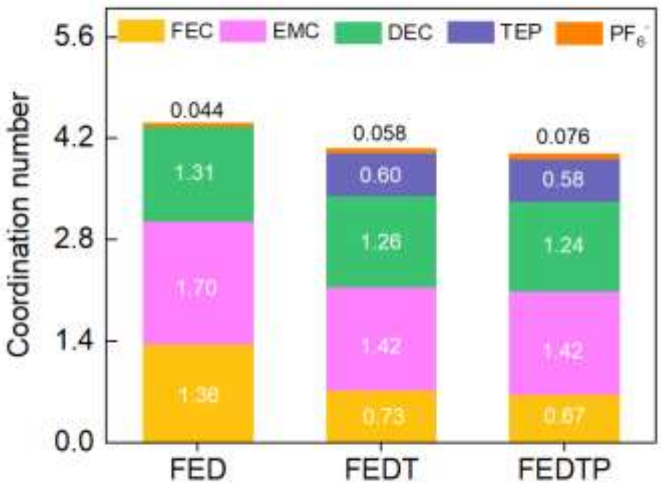

Figure S15. Coordination environment of Li^+ in various electrolytes at 25°C according to the molecular dynamics simulation.

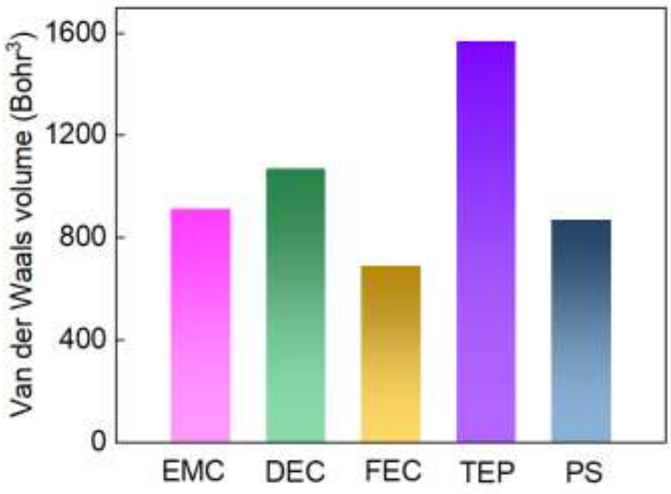

Figure S16. Van der Waals volume of various electrolyte molecules.

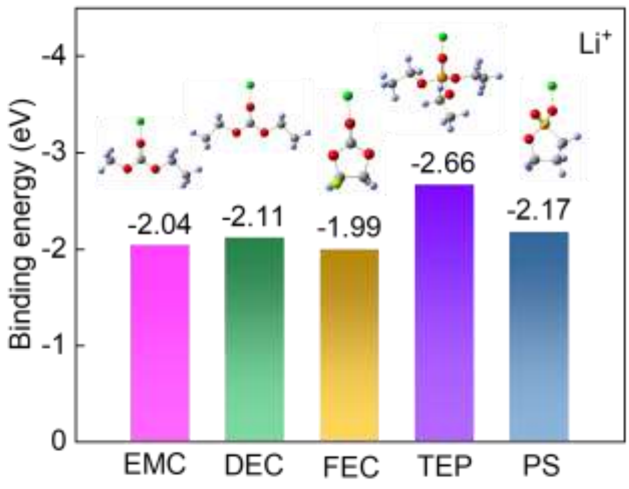

Figure S17. Binding energies of electrolyte molecules with Li^+.

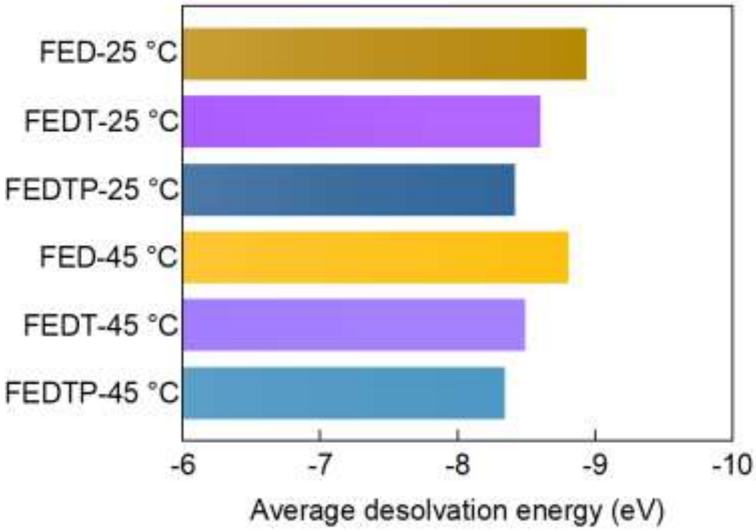

Figure S18. Average Li^+ de-solvation energies of various electrolytes at 25°C or 45°C.

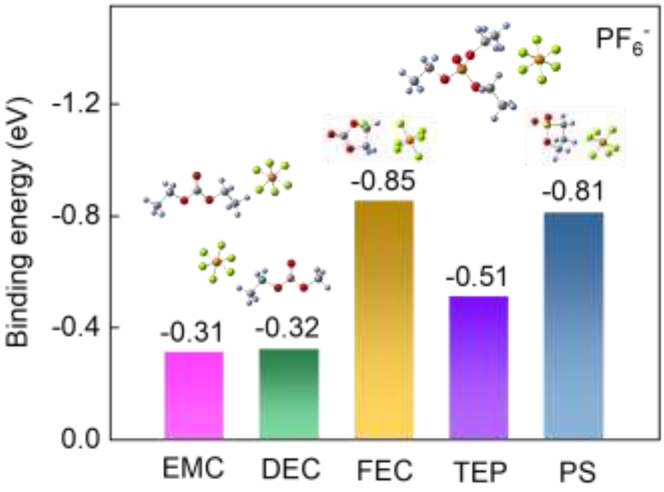

Figure S19. Binding energies of electrolyte molecules with PF_6^-.

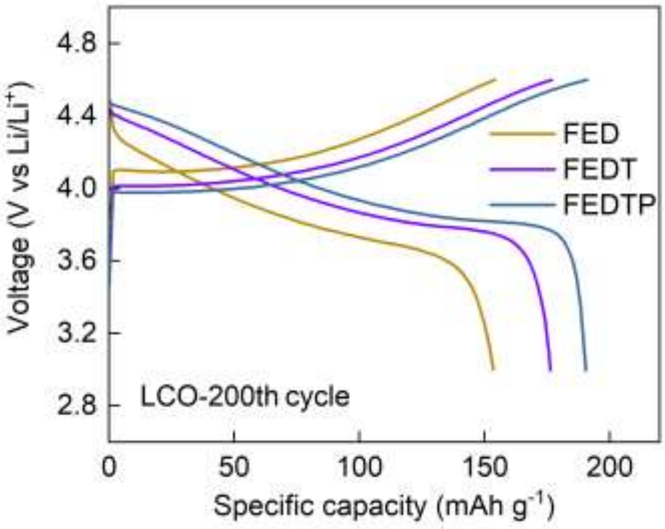

Figure S20. Voltage profiles of LCO cathodes with various electrolytes after 200 cycles at 45°C.

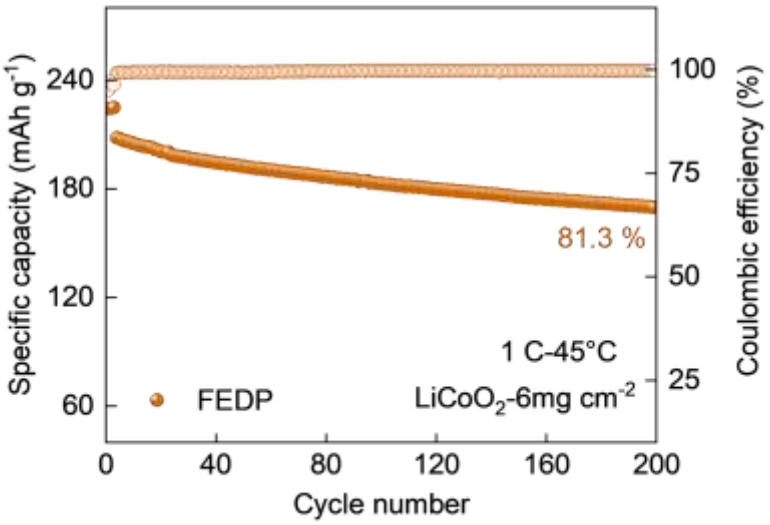

Figure S21. Galvanostatic cycling performance and coulombic efficiency of LCO cathodes with FEDP electrolyte (FED electrolyte with 1 vol% PS) at a rate of 0.2 C for the first 3 cycles and 1C for the subsequent cycles.

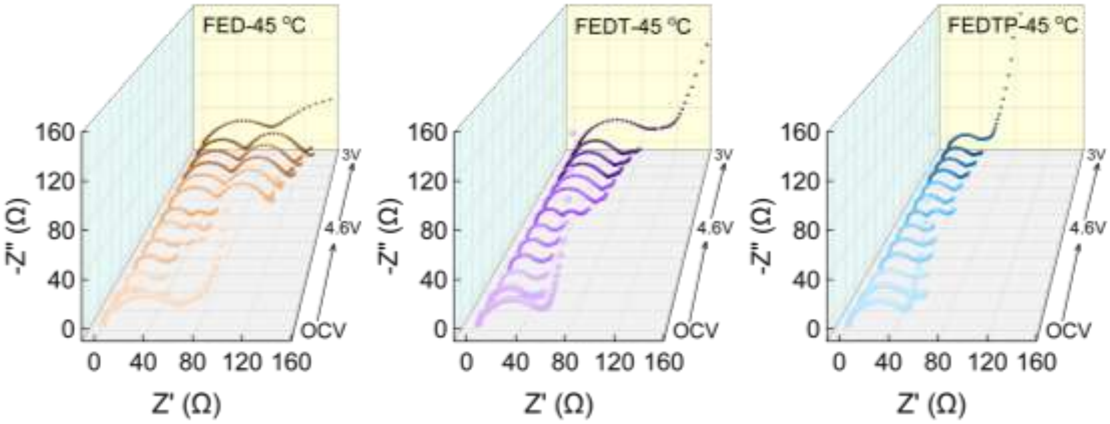

Figure S22. In-*situ* EIS of LCO cathodes with various electrolytes at the 1st cycle under 45°C.

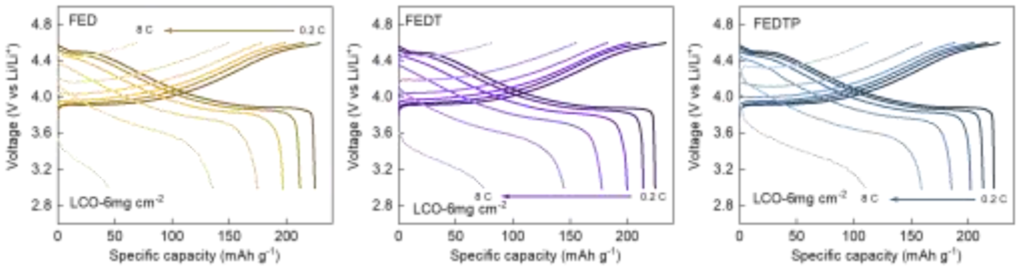

Figure S23. Voltage profiles of LCO cathodes with different current density in various electrolytes.

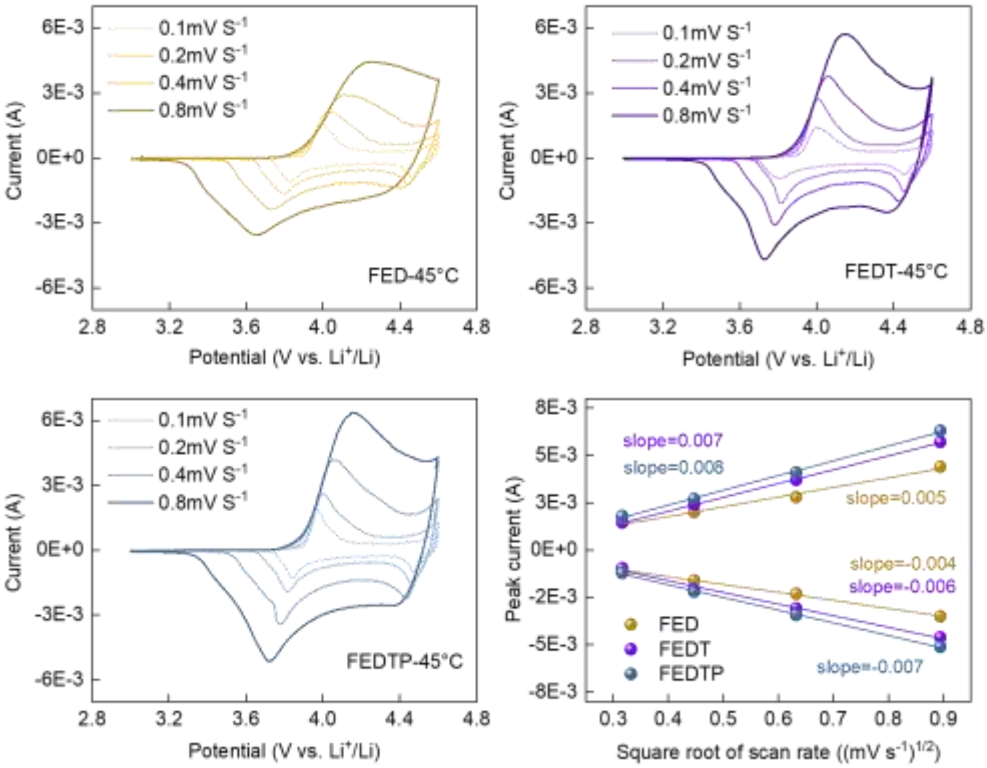

Figure S24. CV curves of LCO cathodes with various electrolytes at different scan rates and the corresponding linear relationship between the peak current and square root of scan rate.

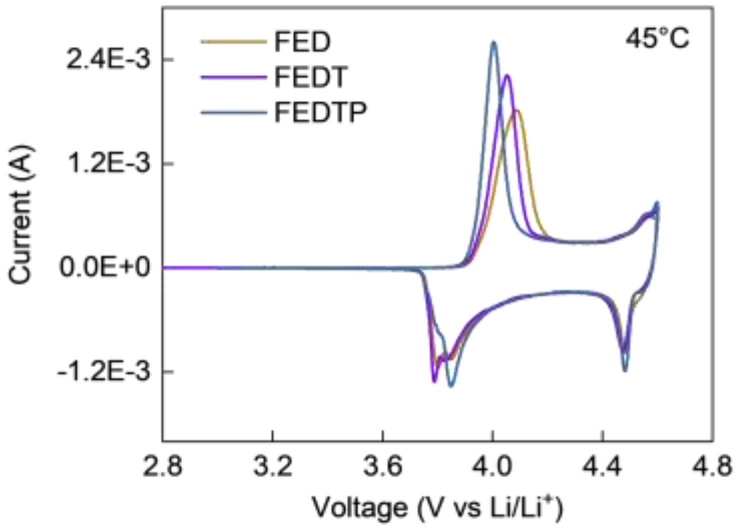

Figure S25. CV curves of the first cycle for LCO cathodes with various electrolytes at 0.1 mV s^{-1}.

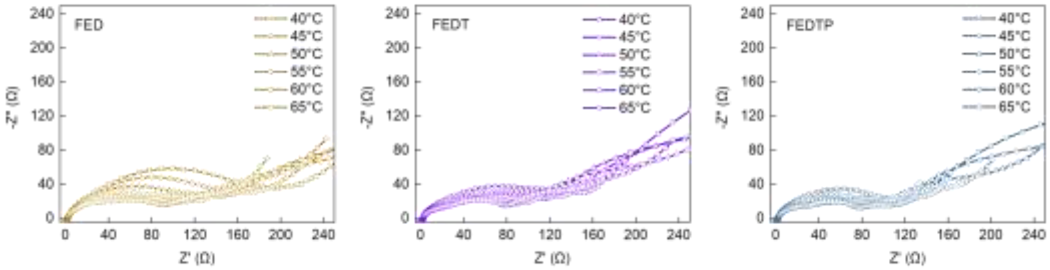

Figure S26. EIS of LCO cathodes with various electrolytes at various temperatures.

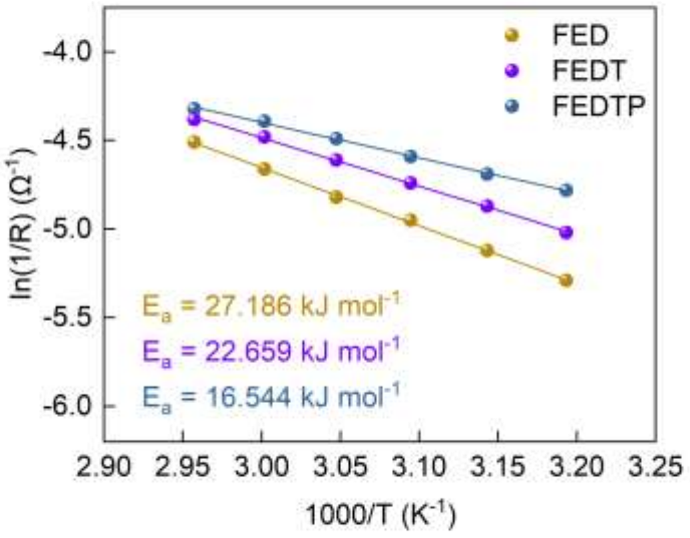

Figure S27. Arrhenius behavior and comparison of activation energies for R_{CEI} with various electrolytes.

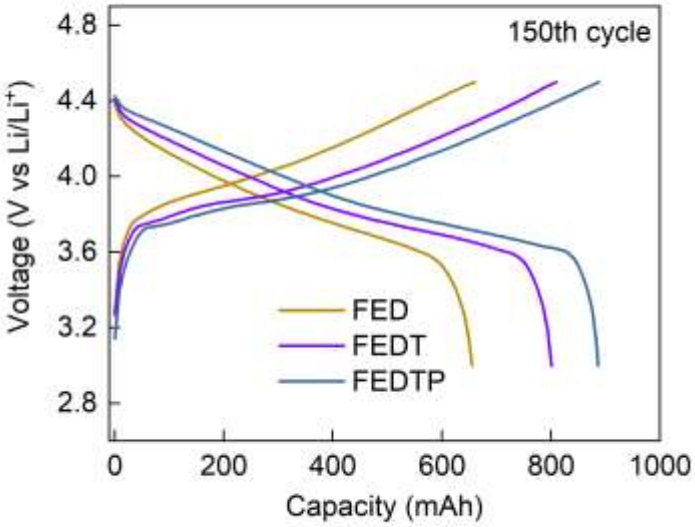

Figure S28. Voltage profiles of pouch cells with various electrolytes after 150 cycles at 45°C.

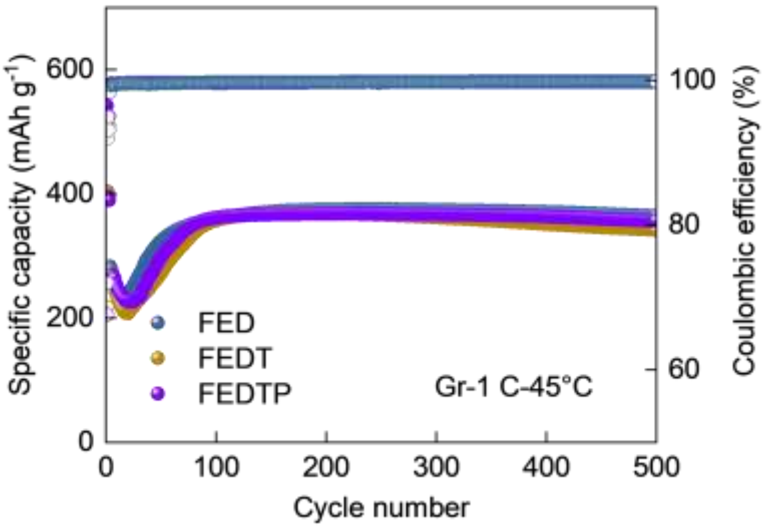

Figure S29. Galvanostatic cycling performance and coulombic efficiency of graphite (Gr) anodes with various electrolytes at a rate of 0.2 C for the first 3 cycles and 1 C for the subsequent cycles.

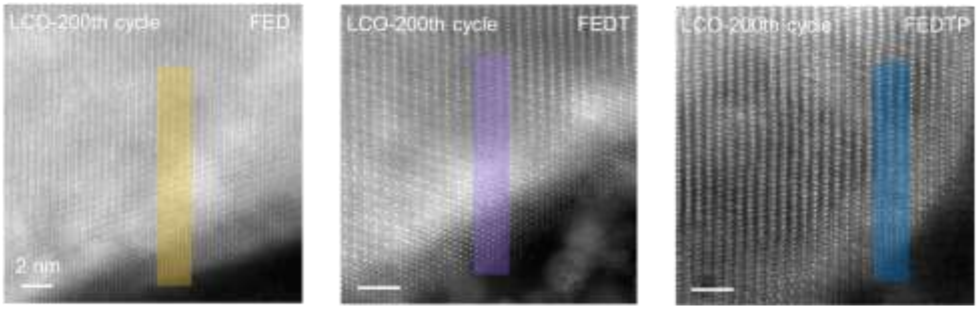

Figure S30. HAADF-STEM images of LCO cathodes with various electrolytes after 200 cycles at 45°C (EELS spectra are obtained from the selected areas).

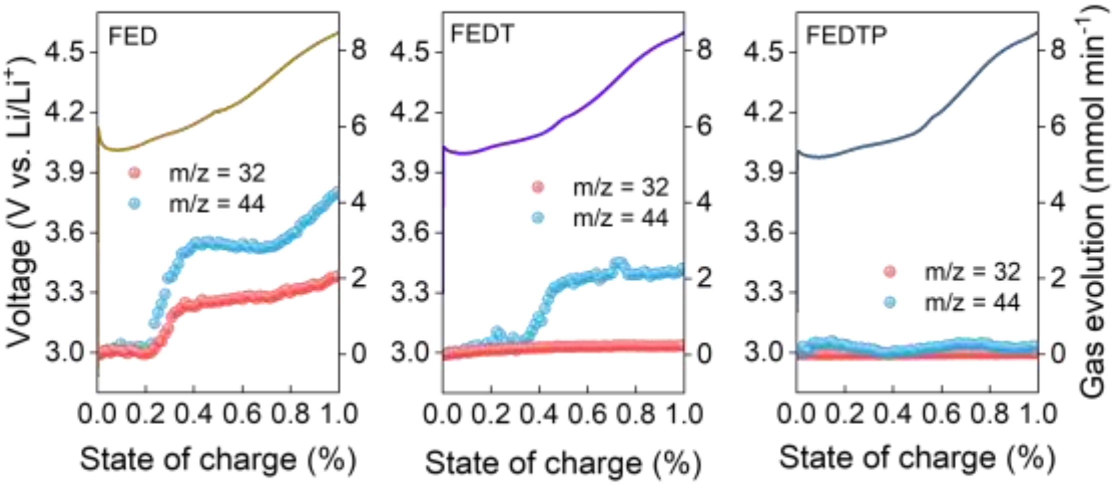

Figure S31. In-*situ* DEMS test of LCO cathodes with various electrolytes at a rate of 0.3 C by performing charging from 3.0 to 4.6 V at 45°C.

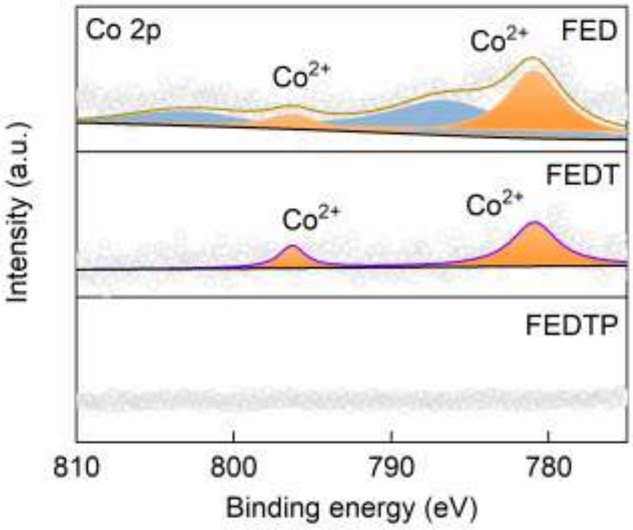

Figure S32. Co 2p XPS spectra of Li metal for LCO cathodes with various electrolytes after 200 cycles at 45°C.

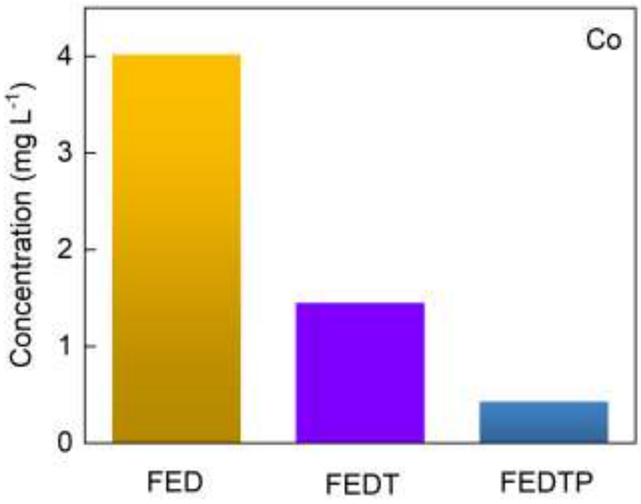

Figure S33. Content of Co ions in separators of LCO cells with various electrolytes after 200 cycles at 45°C.

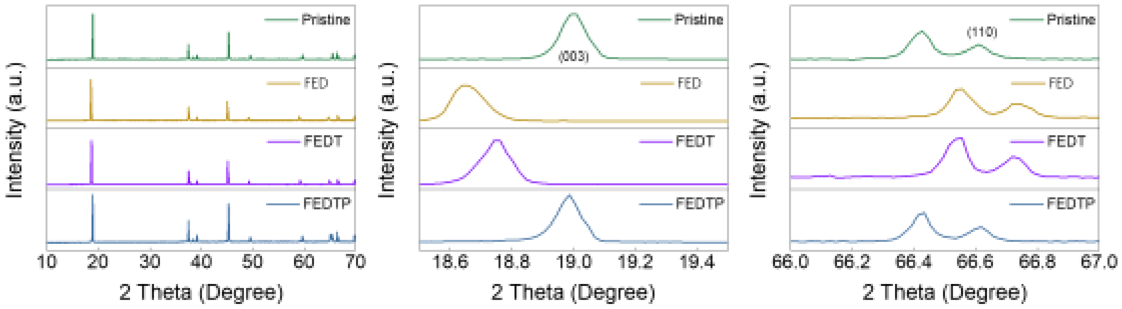

Figure S34. XRD patterns of LCO cathodes before cycling and LCO cathodes various electrolytes after 200 cycles at 45°C.

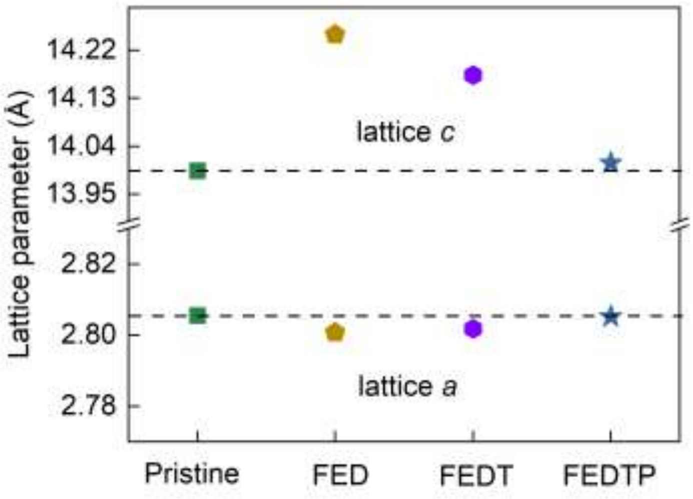

Figure S35. Lattice parameters of LCO cathodes after 200 cycles at 45°C calculated from XRD patterns.

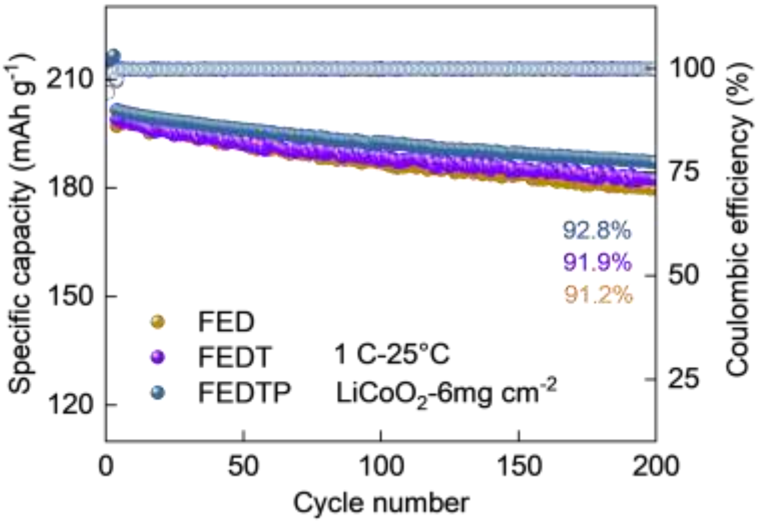

Figure S36. Galvanostatic cycling performance and coulombic efficiency of LCO cathodes with various electrolytes at a rate of 0.2 C for the first 3 cycles and 1 C for the subsequent cycles.

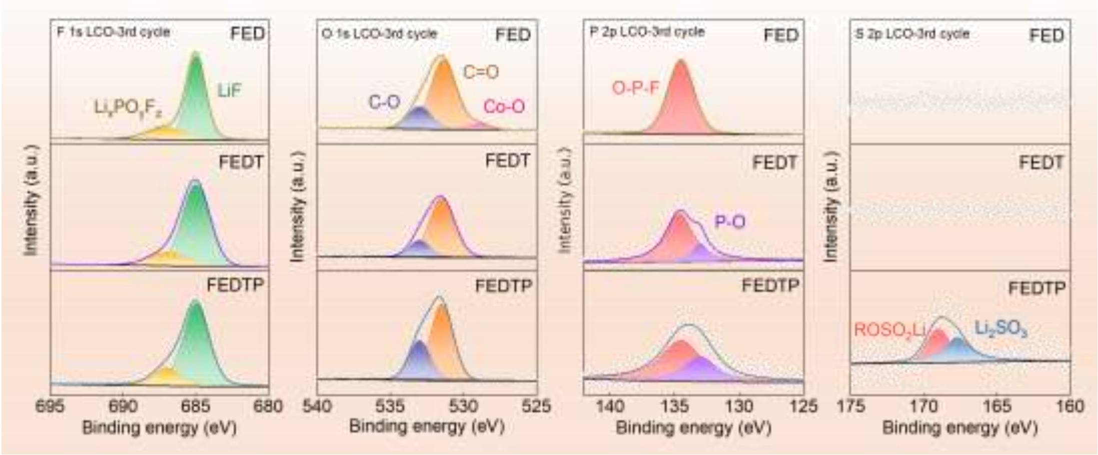

Figure S37. F 1s, P 2p, O 1s and S 2p X-ray photoelectron spectra of LCO cathodes with various electrolytes after 3 cycles at 45°C.

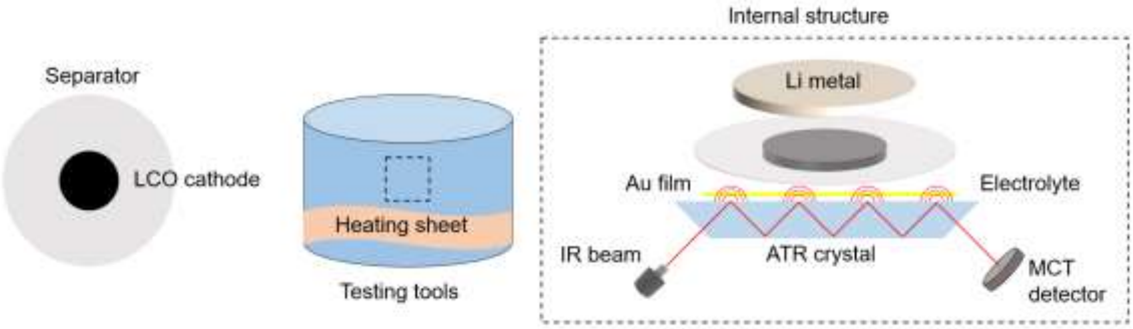

Figure S38. Schematic illustration of surface-enhanced in-*situ* FTIR test.

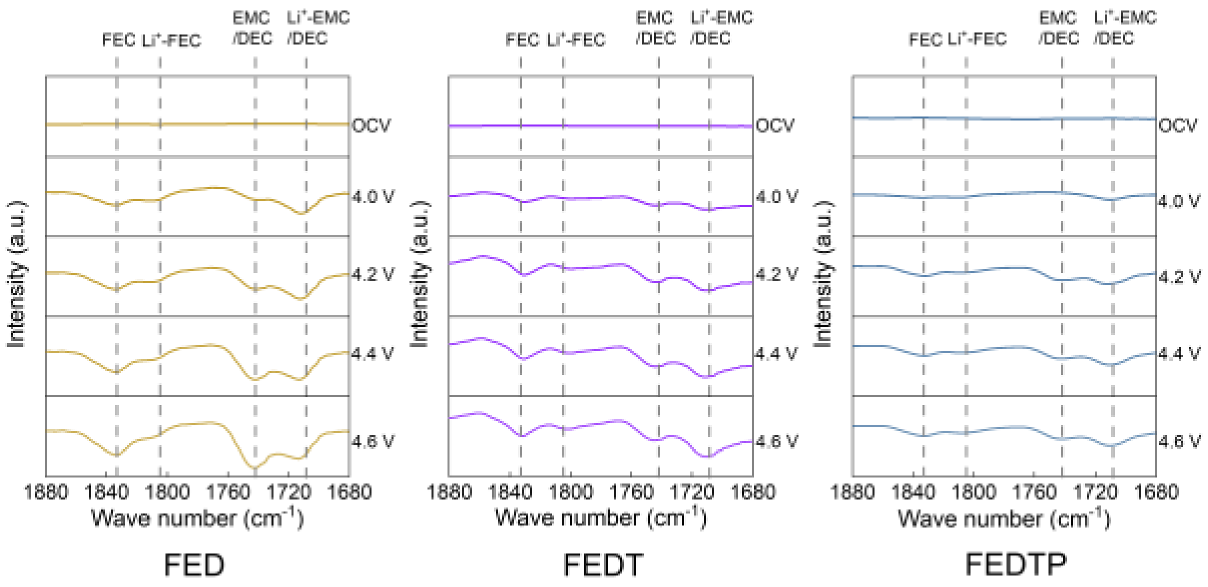

Figure S39. Surface-enhanced in-*situ* FTIR spectra of LCO cathodes with various electrolytes in the first charge at 45°C.

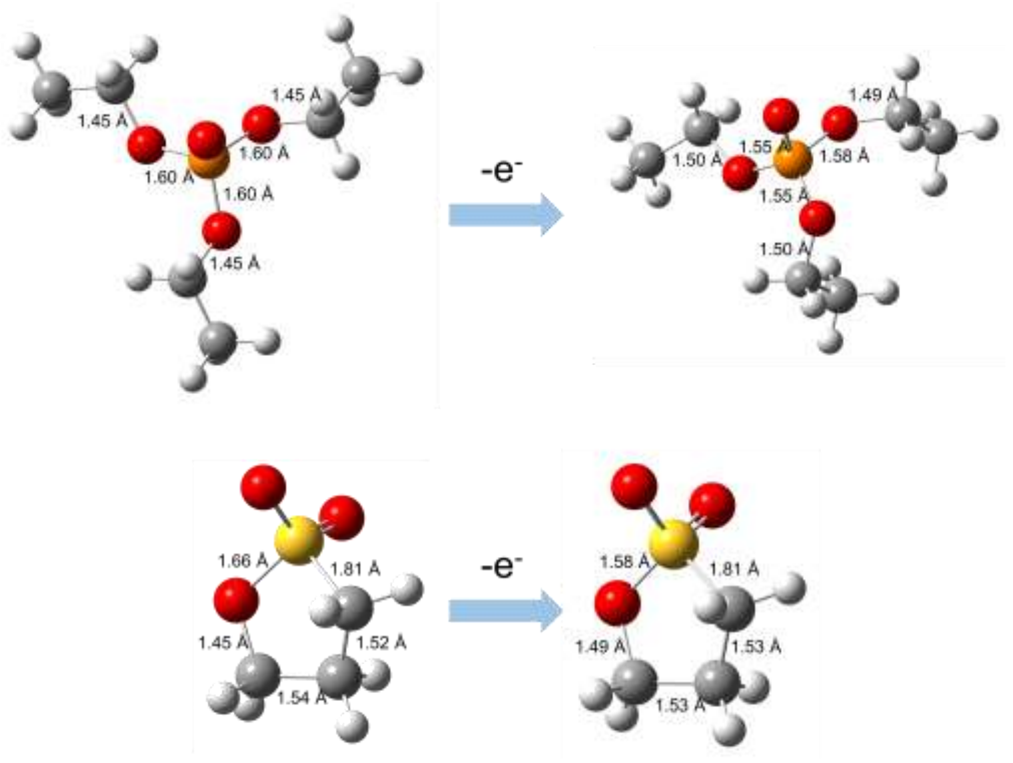

Figure S40. Oxidation process of TEP and PS molecules based on DFT calculation (the number in Figure is the length of chemical bond).

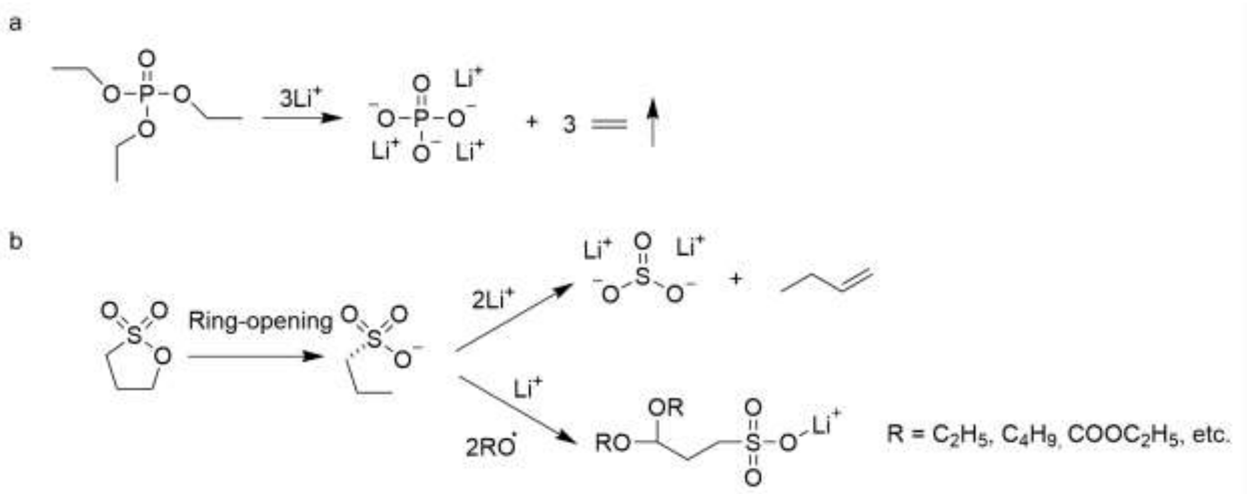

Figure S41. Proposed decomposition paths of (a) TEP and (b) PS.

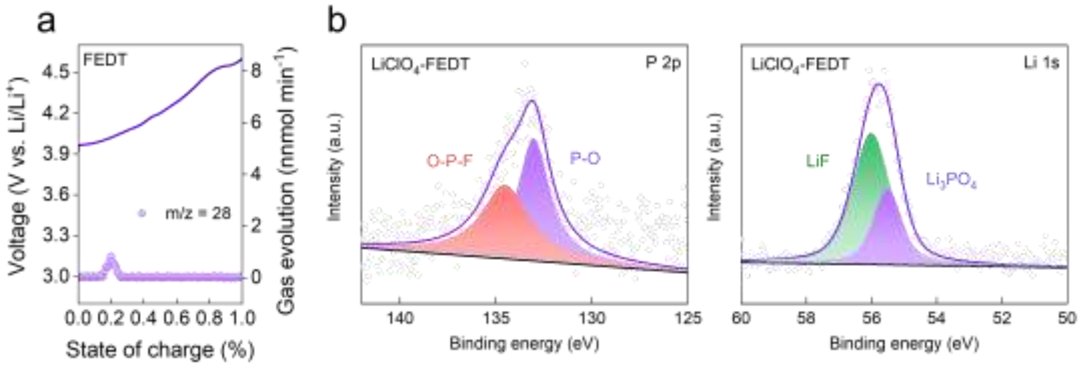

Figure S42. (a) In-*situ* DEMS test of LCO cathodes with FEDT electrolyte; (b) P 2p and Li 1s X-ray photoelectron spectra LCO cathodes with FEDT electrolyte (using $LiClO_4$ as Li-salt) after 3 cycles at 45°C.

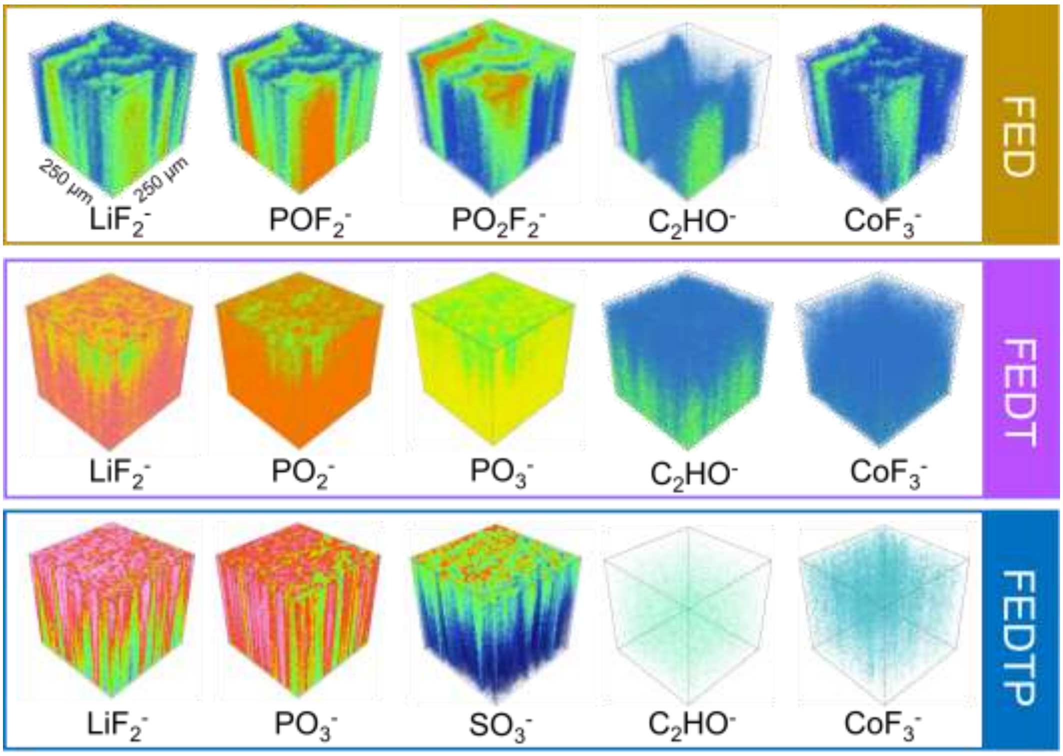

Figure S43. Time of flight secondary-ion mass spectrum (TOF-SIMS) three-dimensional distributions (right) on LCO cathode with various electrolytes after 200 cycles at 45°C (the higher color saturation represents the higher content and the area is 250 μm × 250 μm).

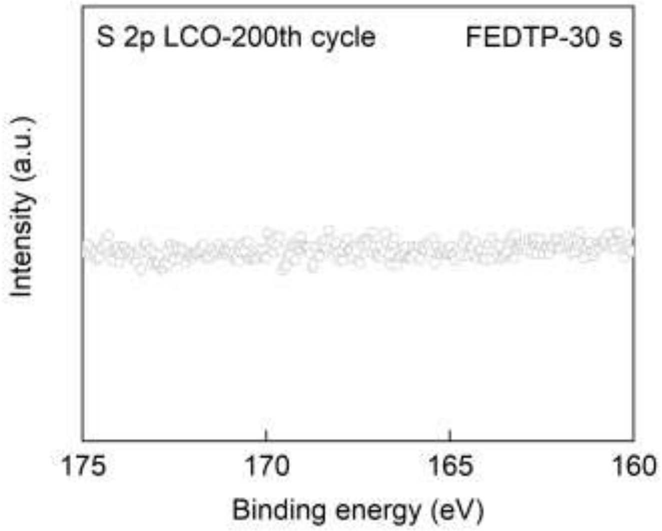

Figure S44. S 2p X-ray photoelectron spectra of LCO cathodes with FEDTP electrolytes after 200 cycles at 45°C.

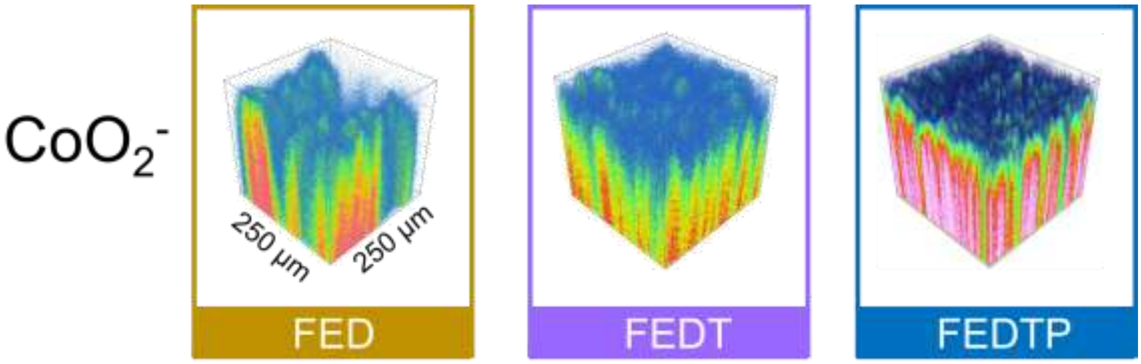

Figure S45. Time of flight secondary-ion mass spectrum (TOF-SIMS) three-dimensional distributions on LCO cathode with various electrolytes after 200 cycles at 45°C (the higher color saturation representing the higher content).

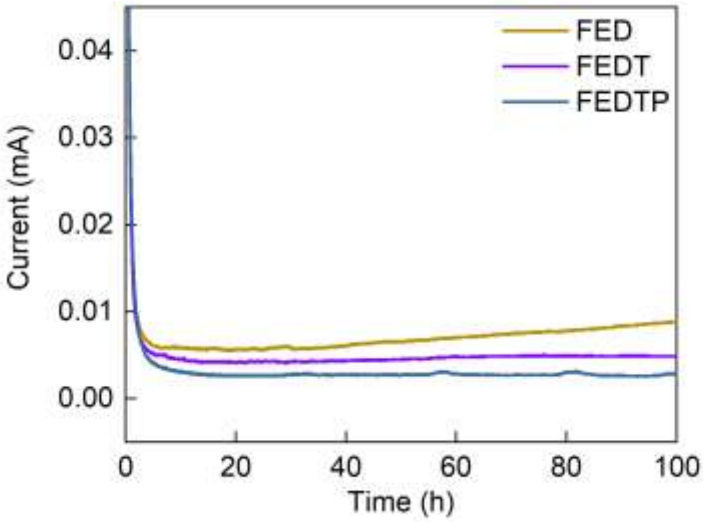

Figure S46. Floating charge leakage current curves of LCO cathodes with various electrolytes at 0.2 C.

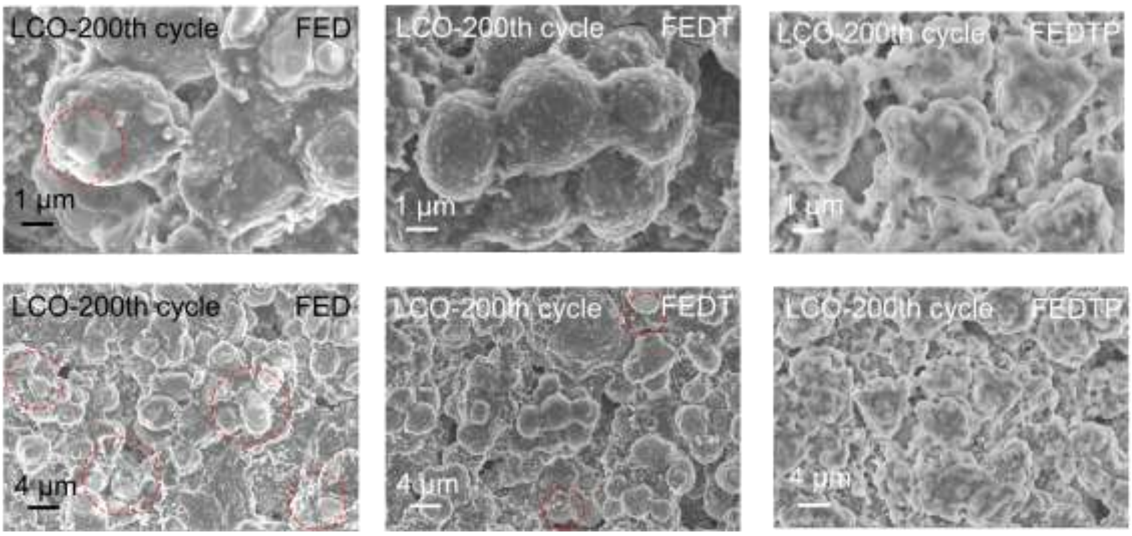

Figure S47. Top-view scanning electron microscopy images of LCO cathodes with various electrolytes after 200 cycles at 45°C.

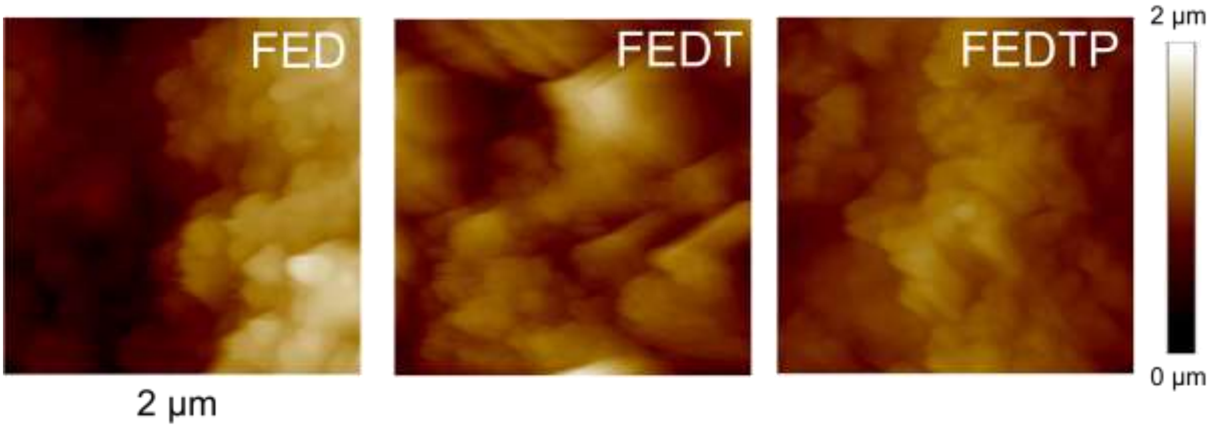

Figure S48. Surface morphology of CEI on the LCO cathodes after 200 cycles at 45°C with various electrolytes measured *via* AFM (2 μm × 2 μm).

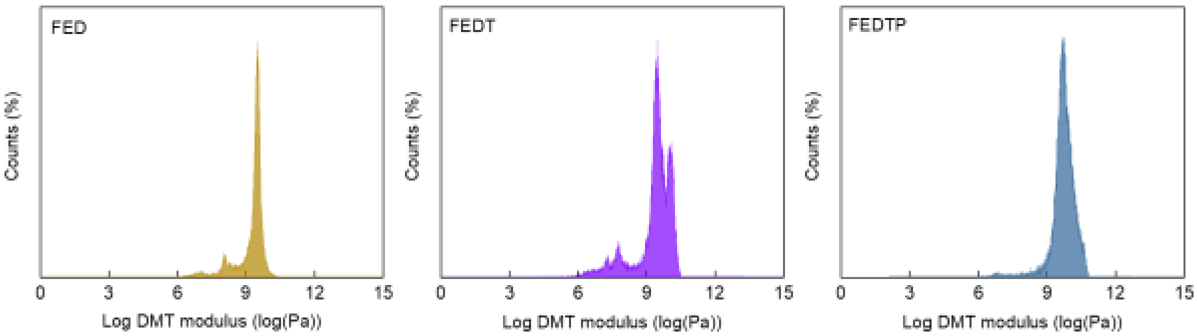

Figure S49. Log DMT modulus distribution of CEI on the LCO cathodes after 200 cycles at 45°C with various electrolytes measured *via* AFM (2 μm × 2 μm).

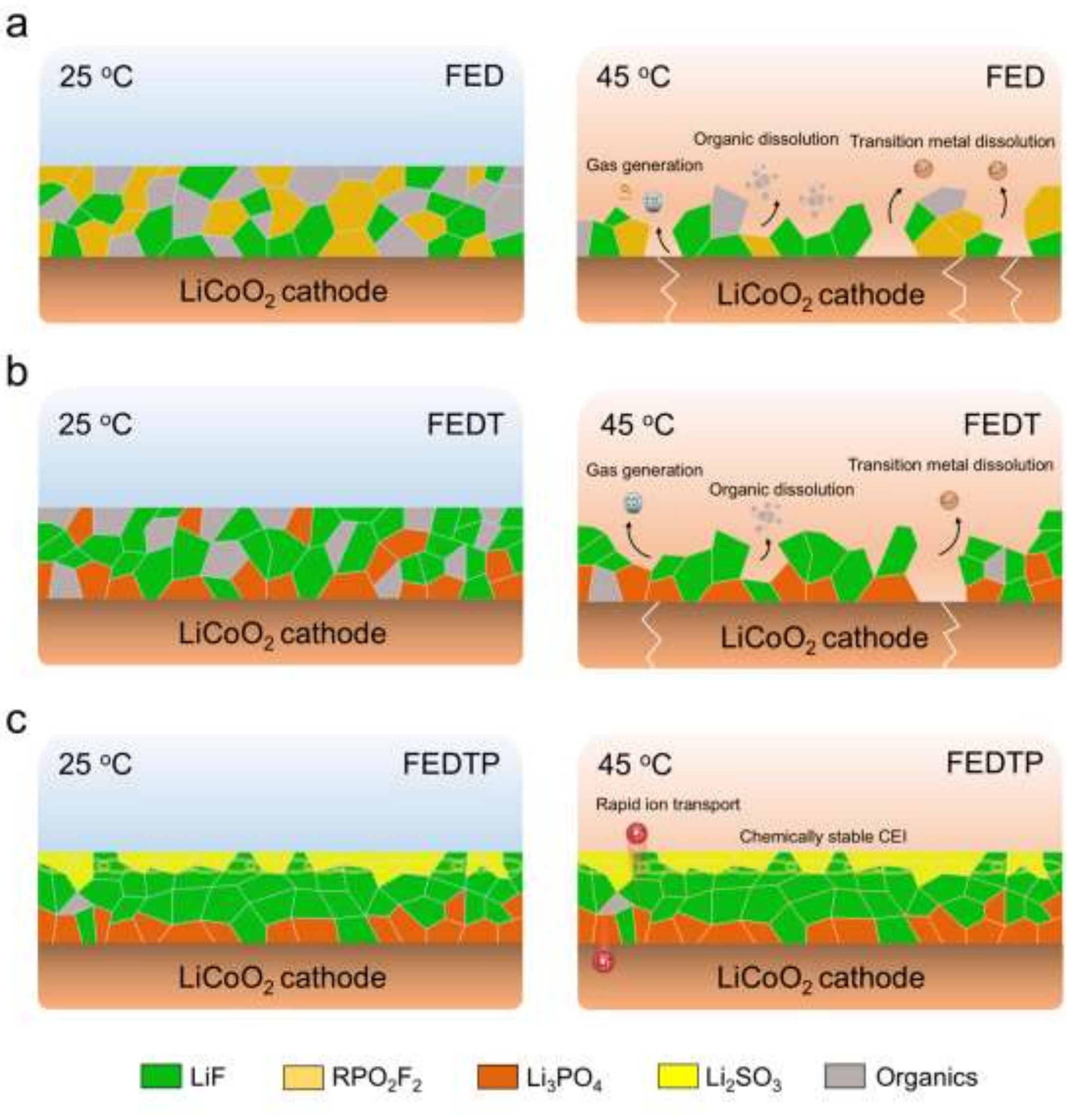

Figure S50. Schematic illustration of various electrolyte systems for the surface structure evolution on the LCO cathodes.

References

[1] Huang W, Zhao Q, Zhang M *et al.* Surface design with cation and anion dual gradient stabilizes high-voltage $LiCoO_2$. *Adv Energy Mater* 2022; 12: 2200813.

[2] Li Z, Yi H, Ren H *et al.* Multiple surface optimizations for a highly durable $LiCoO_2$ beyond 4.6 V. *Adv Funct Mater* 2023; 33: 2307913.

[3] Yan Y, Zheng Y, Zhang H *et al.* Blending layered cathode with olivine: an economic strategy for enhancing the structural and thermal stability of 4.65 V $LiCoO_2$. *Adv Funct Mater* 2023; 33: 2304496.

[4] Zhang W, Cheng F, Wang M *et al.* Collective surface enabling an ultralong life of $LiCoO_2$ at high voltage and

elevated temperature. *Adv Funct Mater* 2023; 33: 2304008.

[5] Wang Y, Zhang Q, Xue ZC *et al.* An in situ formed surface coating layer enabling $LiCoO_2$ with stable 4.6 V high-voltage cycle performances. *Adv Energy Mater* 2020; 10: 2001413.

[6] Lan X, Yang S, Meng T *et al.* A multifunctional electrolyte additive with solvation structure regulation and electrode/electrolyte interface manipulation enabling high-performance Li-ion batteries in wide temperature range. *Adv Energy Mater* 2023; 13: 2203449.

[7] Ren J, Tang Y, Li W *et al.* Enabling high-performance 4.6 V $LiCoO_2$ in a wide temperature range via a synergetic strategy. *EcoMat* 2023; 5: e12344.

[8] Sun Z, Li F, Ding J *et al.* High-voltage and high-temperature $LiCoO_2$ operation via the electrolyte additive of electron-defect boron compounds. *ACS Energy Lett* 2023; 8: 2478–87.

[9] Yang X, Wang C, Yan P *et al.* Pushing lithium cobalt oxides to 4.7 V by lattice-matched interfacial engineering. *Adv Energy Mater* 2022; 12: 2200197.

[10] Huang H, Li Z, Gu S *et al.* Dextran sulfate lithium as versatile binder to stabilize high-voltage $LiCoO_2$ to 4.6 V. *Adv Energy Mater* 2021; 11: 2101864.

[11] Fan T, Kai W, Harika VK *et al.* Operating highly stable $LiCoO_2$ cathodes up to 4.6 V by using an effective integration of surface engineering and electrolyte solutions selection. *Adv Funct Mater* 2022; 32: 2204972.

[12] Fu A, Zhang Z, Lin J *et al.* Highly stable operation of $LiCoO_2$ at cut-off ≥ 4.6 V enabled by synergistic structural and interfacial manipulation. *Energy Storage Mater* 2022; 46: 406–16.

[13] Fan T, Wang Y, Harika VK *et al.* Highly stable 4.6 V $LiCoO_2$ cathodes for rechargeable li batteries by rubidium-based surface modifications. *Adv Sci* 2022; 9: 2202627.

[14] Fu X, Yang M, Chen L *et al.* Amorphous coating and gradient doping stabilizing $LiCoO_2$ cathodes at 4.7 V and 45 ℃. *Chem Eng J* 2025; 513: 162843.

[15] Zhang SD, Wang J, Grundish NS *et al.* High voltage stable $LiCoO_2$ enabled by surface strengthening and bulk doping. *Mater Today* 2025; 86: 238–46.

[16] Zhang A, Bi Z, Wang G *et al.* Regulating electrode/electrolyte interfacial chemistry enables 4.6 V ultra-stable fast charging of commercial $LiCoO_2$. *Energy Environ Sci* 2024; 17: 3021–31.
